# The Rim15-Endosulfine-PP2A^Cdc55^ Signalling Module Regulates Entry into Gametogenesis and Quiescence *via* Distinct Mechanisms in Budding Yeast

**DOI:** 10.1371/journal.pgen.1004456

**Published:** 2014-06-26

**Authors:** Sourav Sarkar, Jacob Z. Dalgaard, Jonathan B. A. Millar, Prakash Arumugam

**Affiliations:** Division of Biomedical Cell Biology, Warwick Medical School, University of Warwick, Coventry, United Kingdom; The University of North Carolina at Chapel Hill, United States of America

## Abstract

Quiescence and gametogenesis represent two distinct survival strategies in response to nutrient starvation in budding yeast. Precisely how environmental signals are sensed by yeast cells to trigger quiescence and gametogenesis is not fully understood. A conserved signalling module consisting of Greatwall kinase, Endosulfine and Protein Phosphatase PP2A^Cdc55^ proteins regulates entry into mitosis in Xenopus egg extracts and meiotic maturation in flies. We report here that an analogous signalling module consisting of the serine-threonine kinase **R**im15, the **E**ndosulfines Igo1 and Igo2 and the Protein **P**hosphatase PP2A^Cdc55^, regulates entry into both quiescence and gametogenesis in budding yeast. PP2A^Cdc55^ inhibits entry into gametogenesis and quiescence. Rim15 promotes entry into gametogenesis and quiescence by converting Igo1 into an inhibitor of PP2A^Cdc55^ by phosphorylating at a conserved serine residue. Moreover, we show that the Rim15-Endosulfine-PP2A^Cdc55^ pathway regulates entry into quiescence and gametogenesis by distinct mechanisms. In addition, we show that Igo1 and Igo2 are required for pre-meiotic autophagy but the lack of pre-meiotic autophagy is insufficient to explain the sporulation defect of *igo1Δ igo2Δ* cells. We propose that the Rim15-Endosulfine-PP2A^Cdc55^ signalling module triggers entry into quiescence and gametogenesis by regulating dephosphorylation of distinct substrates.

## Author Summary

The fundamental property of a cell is to sense changes in the environment and then respond in a way that maximizes its chances of survival. When diploid budding yeast cells are subjected to complete nutrient starvation they have two possible fates, namely quiescence and gametogenesis. Quiescent cells have reduced rates of transcription and translation and increased stress tolerance. Gametogenesis results in production of haploid spores that can survive for long periods of time. In this paper, we report a signalling module that regulates entry into both quiescence and gametogenesis in budding yeast. The module consists of three molecular components namely a serine-threonine kinase Rim15, a phosphatase PP2A^Cdc55^ and a conserved protein called as endosulfine. PP2A^Cdc55^ negatively regulates entry into gametogenesis and quiescence. Upon nutrient starvation, Rim15 becomes active and phosphorylates endosulfine. This converts endosulfine to an inhibitor of PP2A^Cdc55^ and thereby leading to entry into quiescence and gametogenesis. Remarkably, an analogous module consisting of Greatwall kinase, PP2A-B55δ and endosulfine regulates entry into mitosis in frog egg extracts and meiotic maturation in flies suggesting that this signalling module is highly conserved and co-opted during evolution to control distinct biological processes in different organisms.

## Introduction

The ability of cells to sense deleterious changes in environment and mount an appropriate physiological and metabolic response is essential for cellular survival. Response to nutrition starvation in budding yeast has been an extremely powerful model to study this biological trait [1]. Upon complete nutrient starvation, yeast cells enter either gametogenesis or quiescence. Diploid yeast cells undergo gametogenesis when subjected to nitrogen starvation in the absence of glucose and in the presence of a non-fermentable carbon source. They undergo one round of DNA replication followed by two rounds of nuclear divisions to form 4 haploid spores which can stay dormant for long periods of time. Haploid and diploid cells enter quiescence when subjected to nutrient starvation or when treated with a drug called rapamycin, an inhibitor of the TOR (Target of Rapamycin) signalling pathway. Quiescence (-also referred to as G_0_) is a reversible non-proliferative state characterized by low rates of transcription and translation, increased stress-tolerance, elevated rate of macroautophagy and synthesis of storage carbohydrates (trehalose and glycogen). Many of the G_0_-features like increased macroautophagy, low rates of transcription and translation are also characteristic of quiescent mammalian cells suggesting that the core features of quiescence program are conserved [2], [3]. Ablation of G_0_-entry/exit control mechanisms is frequently linked to either reduced life span (especially in unicellular organisms) or cellular transformation (in multi-cellular organisms) [4], [5].

In budding yeast, entry into quiescence is controlled by the master regulator Rim15, a member of the AGC (named after protein kinase A, G and C families) group of serine-threonine kinases [6]. Activity of Rim15 is controlled by two nutrient signalling pathways namely the Ras/Protein Kinase A (Ras/PKA) and the *T*arget *o*f *R*apamycin *C*omplex 1 (TORC1) pathways. The TORC1 pathway responds to the availability of nitrogen source in the growth medium [2], [5]. In contrast, the Ras/PKA pathway responds to levels of glucose in the growth medium [2], [5]. Both pathways positively regulate cell proliferation in response to nutrient availability and thereby inhibit entry into G_0_. PKA phosphorylates Rim15 at five consensus PKA phosphorylation sites to inhibit its kinase activity and promote its retention in the cytosol [6]. Apart from PKA and TORC1 pathways, Rim15 also integrates signalling from Sch9 kinase (ortholog of mammalian Akt/S6 kinase) and Pho85-Pho80 kinase (phosphate-sensing) pathways [7]. Signalling through TORC1, Sch9 and Pho85/Pho80 pathways phosphorylate Rim15 at Thr-1075 and inhibit its nuclear localization. Nutrient deprivation inhibits signalling through these four pathways which results in dephosphorylation of Rim15 at its five PKA sites and Thr-1075 leading to its activation and translocation to the nucleus. Activated Rim15 stimulates stress-responsive transcription factors Msn2/4 and post-diauxic shift transcription factor Gis1 which in turn activate transcription of several genes required for survival in G_0_. Rim15 phosphorylates endosulfines, a highly conserved family of cAMP regulated phosphoproteins to promote entry into quiescence [8]. Endosulfines, following phosphorylation by Rim15, protect mRNA, which are transcriptionally controlled by Msn2/4 and Gis1, from degradation via the 5′-3′ mRNA decay pathway by inhibiting Dhh1 (decapping activator) and Ccr4 (deadenylation factor) [8].

Entry into gametogenesis in yeast is mainly regulated at the level of transcription of *IME1* which encodes the master transcription factor for early-meiosis genes (EMGs) [9]. Like G_0_, entry into gametogenesis is negatively regulated by TORC1 and Ras/PKA pathways. Ime1 is recruited to promoters of EMGs and activates their transcription [10]. During vegetative growth, a DNA-binding protein Ume6 binds to promoters of EMGs and represses their expression by associating with the Sin3/Rpd3 histone deacetylase and Isw2 chromatin remodelling complexes [11], [12]. Absence of glucose and nitrogen in the medium results in the replacement of Sin3/Rpd3 and Isw2 by Ime1 at the EMG promoter regions [13]. It was proposed that Ime1 activates transcription of EMGs by converting Ume6 from a repressor into an activator [10]. This model was consistent with the observations that Ime1 physically interacts with Ume6 and that cells lacking Ume6 fail to sporulate efficiently [10], [14], [15]. However, this model was disputed by subsequent studies which showed that interaction of Ime1 with Ume6 facilitates Ume6 destruction and meiotic gene induction [16]. Rim15 has been implicated in the removal of histone deacetylase complex from the promoters of EMGs [13] but precisely how this is achieved is not known.

In this paper, we demonstrate that endosulfines are required for entry into gametogenesis and quiescence in budding yeast. Phosphorylation of endosulfine by Rim15 results in its association with the protein phosphatase PP2A^Cdc55^ and inhibition of its phosphatase activity. We show that the Rim15-endosulfine-PP2A^Cdc55^ signalling module regulates entry into quiescence and gametogenesis by distinct mechanisms. We also demonstrate that this signalling module is required for pre-meiotic autophagy which is necessary for gametogenesis in budding yeast. Remarkably a similar signalling module regulates M-phase progression during mitosis and meiosis in higher eukaryotes. In Xenopus egg extracts, the Greatwall kinase phosphorylates α-endosulfine (ENSA) and Arpp19 at a conserved serine residue, which then inhibits PP2A-B55δ to promote entry into mitosis [17], [18]. Depletion of Greatwall kinase and endosulfine in Drosophila leads to mitotic defects suggests that the module regulates entry into mitosis in flies [19], [20]. Inactivation of endosulfine in flies causes a failure in oocyte progression from prophase I to metaphase I indicating that this module regulates entry into M-phase during meiosis [21]. Our results therefore expand the repertoire of functions for this highly conserved signalling module that regulates distinct biological processes in different systems.

## Results

### Endosulfines are required for entry into gametogenesis

**Figure 1:**
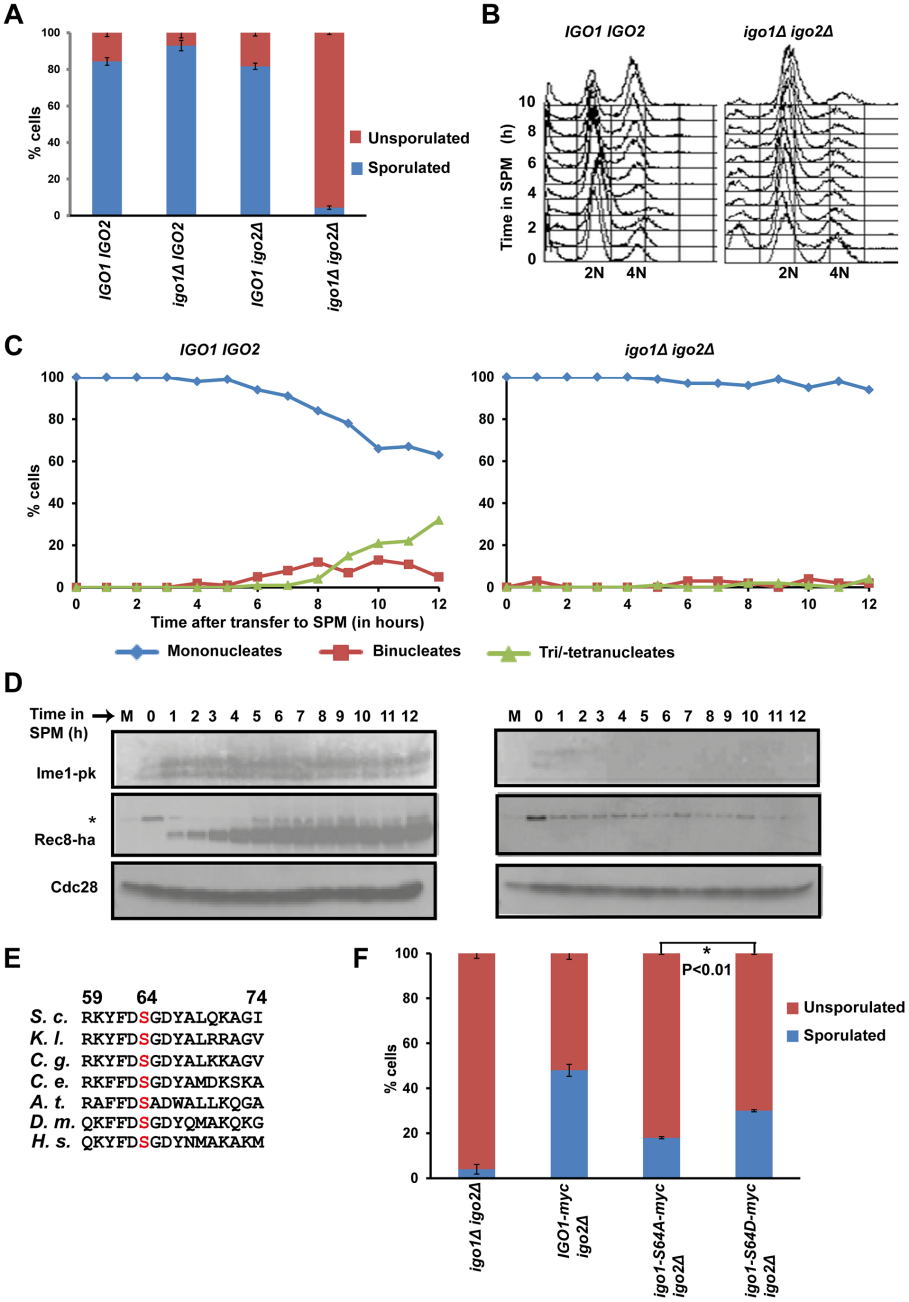
Endosulfines are required for entry into gametogenesis. A) Wild-type, *igo1Δ*, *igo2Δ* and *igo1Δ igo2Δ* cells were incubated for 24 hours on sporulation plates and number of sporulated (includes monad, dyad, Tri-/tetrads) and unsporulated cells were counted using a light microscope. The experiment was repeated 3 times and 200 cells were counted every time for each strain. B) Wild-type and *igo1Δ igo2Δ* cells were transferred to sporulation medium (SPM) and DNA content was measured by flow cytometry. C) Kinetics of nuclear division of cells in B was measured after staining cells with DAPI (n = 200). D) Analysis of expression of early meiotic proteins in cells described in B. Whole-cell extracts of hourly culture in SPM were subjected to western analysis using anti-PK (to detect Ime1), anti-HA (to detect Rec8) and Cdc28 antibody (loading control). The lane indicated as M contains mitotic extracts and * indicate a non-specific band. E) Conserved Rim15/Greatwall kinase site present in endosulfines from *Saccharomyces cerevisiae* (*S.c*), *Kluyveromyces lactis* (*K.l.*), *Candida glabrata* (*C.g*.), *Caenorhabditis elegans* (*C.e.*), *Drosophila melanogaster* (*D.m.*), *Arabidopsis thaliana* (*A.t.*) and humans *(H.s.)* is indicated. F) *igo1Δ igo2Δ* cells and *igo1Δ igo2Δ* cells containing either pRS303-*IGO1-myc8* or pRS303-*IGO1-S64A-myc8* or pRS303-*IGO1-S64D-myc8* were incubated for 24 hours on sporulation plates and number of sporulated (includes monad, dyad, triads/tetrads) and unsporulated cells were counted using a light microscope. Values are expressed as mean ± s.e.m of 3 independent measurements. *P<0.01 (Student's t-test).

Since Rim15 is required for expression of early meiotic genes [22] we examined the function of endosulfine in gametogenesis. Budding yeast has two endosulfines Igo1 and Igo2. We first assessed the ability of wild type, *igo1Δ*, *igo2Δ* and *igo1Δ igo2Δ* strains to sporulate. While wild type, *igo1Δ* and *igo2Δ* strains sporulated with an efficiency of ≥65%, only 3% of *igo1Δ igo2Δ* cells formed spores (Figure 1A). To determine the precise function of endosulfines in spore formation, we induced wild type and *igo1Δ igo2Δ* cells to enter meiosis by transferring them to Sporulation medium (SPM). We examined expression of early meiotic proteins Ime1 and Rec8 (by Western blotting), pre-meiotic DNA replication (flow cytometry) and nuclear division (DAPI staining). Wild type cells replicated their DNA after 5 hours into SPM (Figure 1B), expressed Ime1 and Rec8 (Figure 1D), and underwent two rounds of nuclear division to form tetranucleate spores (Figure 1C). However *igo1Δ igo2Δ* cells failed to express both Rec8 and Ime1, did not undergo pre-meiotic DNA replication and remained mononucleate even after 12 hours into SPM (Figure 1B–1D). These results indicate that endosulfines are required for entry into gametogenesis in budding yeast. Induction of sporulation [23] involves arresting cells in stationary phase by growth in nutrient medium contacting acetate as a carbon source for 16 hours. To rule out the possibility that the failure of endosulfine mutant cells to sporulate was due to their inability to exit from stationary phase, we induced logarithmically growing wild type and *igo1Δ igo2Δ* cells to enter gametogenesis. Wild type but not *igo1Δ igo2Δ* cells underwent pre-meiotic DNA replication and spore formation (Figure S1), indicating that endosulfines are required for entry into gametogenesis. The spores formed in *igo1Δ igo2Δ* cells at a low frequency had viabilities similar to wild type spores (data not shown) suggesting that endosulfines are required for efficient entry into gametogenesis but not for rest of the sporulation program.

### Phosphorylation of Igo1 at S64 is required for efficient entry into gametogenesis

Phosphorylation of endosulfine at a conserved serine residue (Figure 1E) by Greatwall kinase is required for entry into mitosis in Xenopus egg extracts [17], [18]. Phosphorylation at the corresponding Serine residue (Serine-64) in budding yeast Igo1 by Rim15 kinase is required for entry into G_0_ [8]. To test whether phosphorylation at S-64 also regulates entry into gametogenesis, we tested the ability of phospho-inhibitory *igo1-S64A* mutant to sporulate. About 50% of *igo1Δ igo2Δ* cells expressing wild type Igo1 sporulated in comparison to just 2% of control *igo1Δ igo2Δ* cells. In contrast, only 10% of *igo1Δ igo2Δ* cells expressing Igo1-S64A sporulated (Figure 1F). The sporulation efficiency of *igo1Δ igo2Δ* cells expressing the phospho-mimetic mutant Igo1-S64D was 1.7 fold more than that of Igo1-S64A expressing *igo1Δ igo2Δ* cells (Figure 1F). This effect of S64D mutation on sporulation efficiency was independent of Rim15 function (Figure S2). These results suggest that phosphorylation of Igo1 at Serine-64 by Rim15 is required for efficient entry into gametogenesis.

Phosphorylation of Igo1 at Serine-64 occurs at a constant level during the mitotic cell cycle [24]. To examine the phosphorylation of Igo1 at Serine-64 during entry into gametogenesis, we induced *IGO1-myc8* and *igo1-S64A-myc8* cells to enter gametogenesis by transferring them to SPM. Analysis of DNA content by flow cytometry indicated that pre-meiotic DNA replication was initiated after 3 hours into SPM and completed by 5 hours in both strains (Figure S3B). We prepared whole cell extracts and analysed electrophoretic mobility of Igo1 by Phos-tag affinity gel electrophoresis and SDS-PAGE. Phos-tag specifically retards the mobility of phosphoproteins [25]. We observed a phos-tag dependent mobility shift of wild type Igo1 but not Igo1-S64A. This upshifted band in wild type cells was present before transfer to SPM and was detectable up to 2 hours after transfer (Figure S3A). As expression of early meiotic genes like Ime1 and Rec8 is detectable even after 1 h in SPM (Figure 1C), we conclude that Igo1 is phosphorylated at S-64 during entry into gametogenesis but dephosphorylated subsequently.

Endosulfine contains a conserved protein kinase A site RK/RXS/T at its C-terminus (Figure S4A). Since PKA inhibits entry into gametogenesis, we reasoned that phosphorylation at this site might have an opposite effect to that mediated by Rim15 phosphorylation of S-64. However replacement of the Serine-105 in Igo1 with alanine or aspartate did not affect sporulation (Figure S4B).

### Depletion/absence of the PP2A regulatory subunit Cdc55 suppress the gametogenesis- and G_0-_ entry defects of endosulfine mutant cells

**Figure 2:**
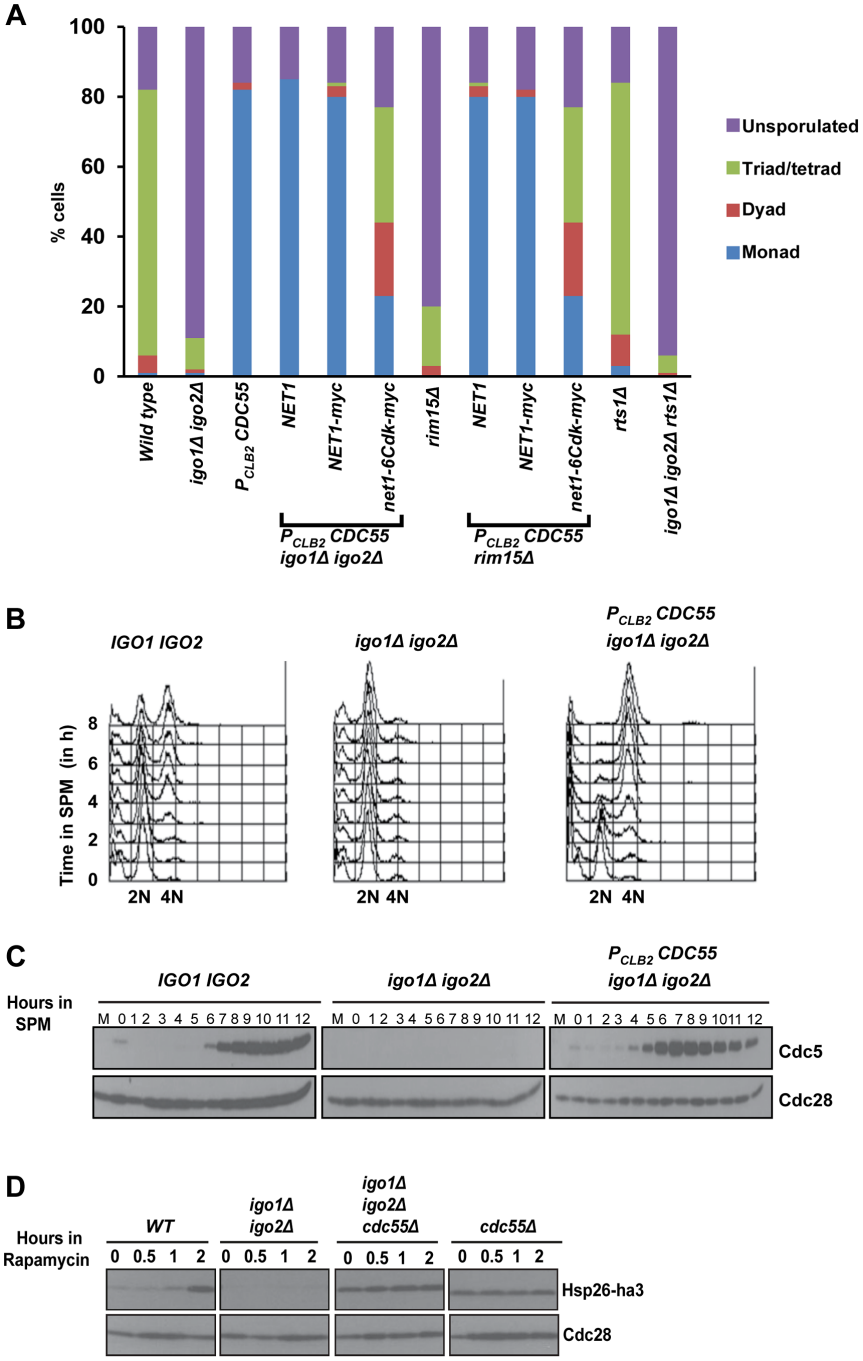
Absence of Cdc55 suppresses the gametogenesis- and quiescence- entry defect of endosulfine mutants. A) Wild-type, *igo1Δ igo2Δ*, *P_CLB2_CDC55* and *igo1Δ igo2Δ P_CLB2_CDC55*, *igo1Δ igo2Δ P_CLB2_CDC55 net1-6Cdk*, *igo1Δ igo2Δ P_CLB2_CDC55 NET1*, *rim15Δ*, *rim15Δ P_CLB2_CDC55*, *rim15Δ P_CLB2_CDC55 net1-6Cdk*, *rim15Δ P_CLB2_CDC55 NET1*, *rts1Δ* and *igo1Δ igo2Δ rts1Δ* cells were incubated on sporulation plates for 24 hours and number of sporulated (includes monad, dyad, triads/tetrads) and unsporulated cells were counted using a light microscope. B) Wild-type, *igo1Δ igo2Δ* and *igo1Δ igo2Δ P_CLB2_CDC55* cells were induced to enter meiosis by transferring them to SPM. Pre-meiotic DNA replication in the cultures was assayed by flow cytometry. C) Analysis of expression of Cdc5. Whole-cell extracts of hourly culture in SPM was prepared by TCA method. Protein samples were run on 10% SDS-PAGE, transferred to nitrocellulose membrane and probed with anti-Cdc5 and anti-Cdc28 antibody respectively. D) Wild-type, *igo1Δ igo2Δ*, *cdc55Δ* and *igo1Δ igo2Δ cdc55Δ* cells expressing Hsp26-ha3 were grown to log phase at 30°C, rapamycin (final concentration 200 ng/ml) was added to each culture and samples were collected at indicated times. Total cell extracts were prepared by TCA method. Proteins were run on 12% SDS-PAGE, transferred to nitrocellulose membrane and probed with anti-HA and anti-Cdc28 antibodies.

Phosphorylated endosulfine promotes entry into mitosis in Xenopus egg extracts by inhibiting the Cdk-antagonizing protein phosphatase PP2A-B55δ [17], [18]. We have demonstrated that *P_CLB2_CDC55* cells which express *CDC55* from the mitosis-specific promoter *P_CLB2_*, fail to undergo meiotic nuclear divisions and form monads [23]. The meiotic nuclear division defect of *P_CLB2_CDC55* cells can be suppressed by *net1-6Cdk*, a mutant allele encoding the nucleolar protein Net1 lacking 6 Cdk recognition sites [23]. We also noted that *P_CLB2_CDC55* cells underwent pre-meiotic DNA replication earlier than wild type cells [23] suggesting that PP2A^Cdc55^ might negatively regulate entry into gametogenesis. We therefore investigated whether budding yeast proteins Rim15, endosulfine and PP2A^Cdc55^ regulate entry into gametogenesis and G_0_. If PP2A^Cdc55^ and Rim15/endosulfines play opposing roles in entry into gametogenesis and endosulfines promote entry into gametogenesis only by antagonising PP2A^Cdc55^ , we reasoned that inactivation of PP2A^Cdc55^ might suppress the sporulation defect of *igo1Δ igo2Δ* and *rim15Δ* cells. While 80% of wild type cells formed spores, only about 10% and 18% of *igo1Δ igo2Δ* and *rim15Δ* cells respectively, did. Remarkably *igo1Δ igo2Δ* and *rim15Δ* cells carrying a meiotic-null allele of *CDC55* (*P_CLB2_CDC55)* formed monads (75%) like *P_CLB2_CDC55* cells (Figure 2A). Crucially, combining *net1-6Cdk* with *P_CLB2_CDC55 igo1Δ igo2Δ* and *P_CLB2_CDC55 rim15Δ* cells resulted in efficient formation of tetrads (Figure 2A). The ability of *P_CLB2_CDC55* to suppress *igo1Δ igo2Δ* was specific as deletion of a gene encoding an alternative PP2A regulatory subunit Rts1 had no effect on sporulation efficiency of *igo1Δ igo2Δ* cells (Figure 2A).

To confirm suppression of *igo1Δ igo2Δ* by *P_CLB2_CDC55* we induced wild type, *igo1Δ igo2Δ* and *igo1Δ igo2Δ P_CLB2_CDC55* cells to enter meiosis by transferring them to SPM. Wild type cells completed pre-meiotic DNA replication after 4 hours (Figure 2B), and expressed Cdc5 (a marker for mid-meiosis) after 7 hours in SPM (Figure 2C). In contrast, *igo1Δ igo2Δ* cells did not initiate DNA replication (Figure 2B) and failed to express Cdc5 even after 12 hours in SPM (Figure 2C). Crucially *igo1Δ igo2Δ P_CLB2_CDC55* cells completed pre-meiotic DNA replication (3–4 hours) and expressed Cdc5 (5–6 hours) (Figure 2B–C). These results indicate that PP2A^Cdc55^ and Rim15/endosulfine play opposing roles in regulating entry into gametogenesis.

We then determined whether PP2A^Cdc55^ also negatively regulates entry into quiescence. Wild type, *igo1Δ igo2Δ*, *cdc55Δ* and *igo1Δ igo2Δ cdc55Δ* cells were treated with rapamycin and entry into G_0_ was monitored by assaying expression of Hsp26, a gene that is specifically induced during entry into G_0_ [8]. While wild type cells induced expression of Hsp26 after 2 hours following rapamycin treatment, the *igo1Δ igo2Δ* cells failed to express Hsp26 (Figure 2D). Crucially, both *cdc55Δ* cells and *igo1Δ igo2Δ cdc55Δ* cells expressed Hsp26 even in the absence of rapamycin treatment. These results indicate that the Rim15-endosulfine-PP2A^Cdc55^ pathway regulates entry into gametogenesis and quiescence in budding yeast.

### Phosphorylation of Igo1 at S64 by Rim15 converts it into an inhibitor of PP2A^Cdc55^

**Figure 3:**
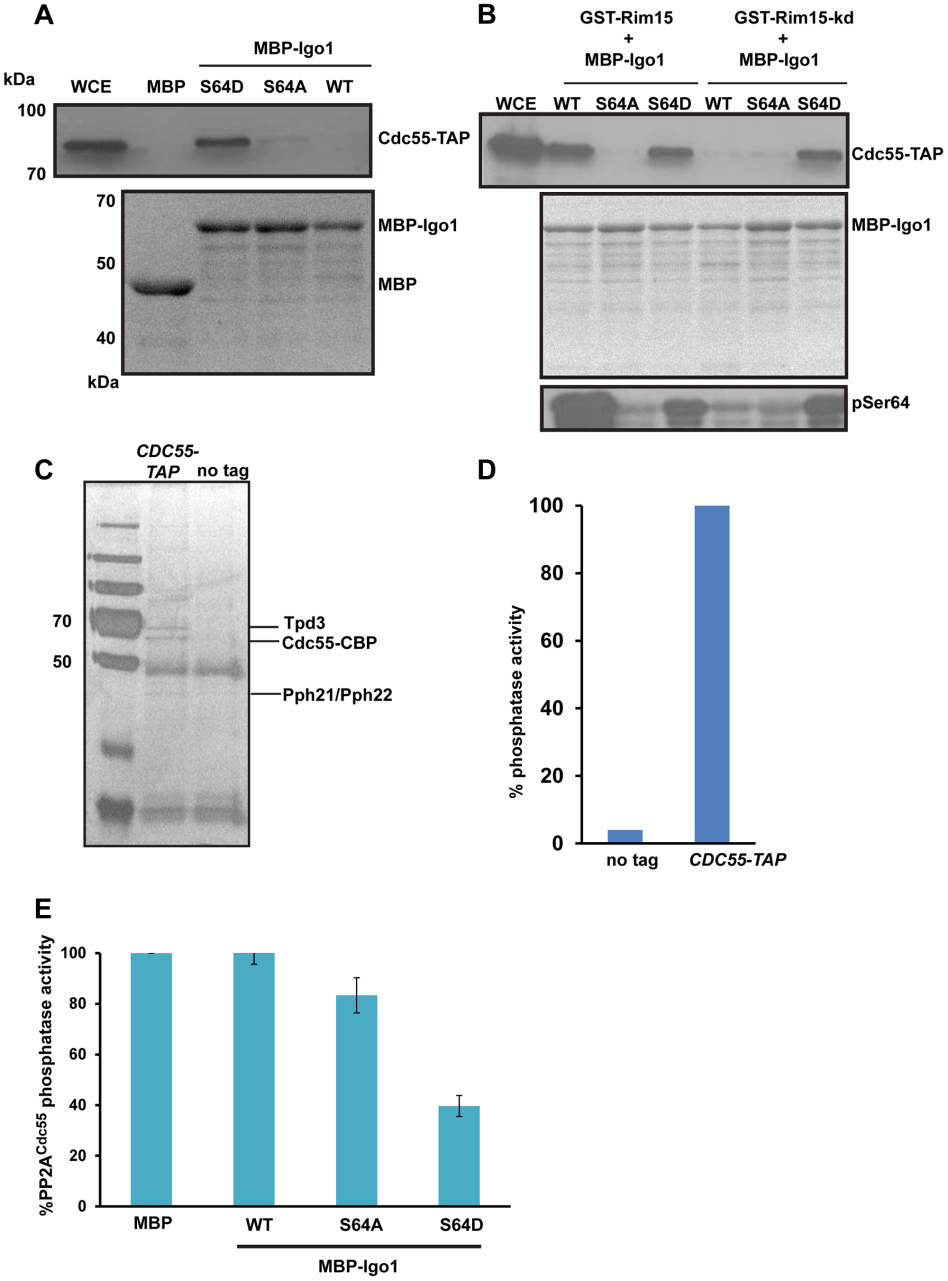
Igo1 associates and inhibits the phosphatase activity of PP2A^Cdc55^ in a phosphorylation dependent manner. A) Either MBP or MBP fused to Igo1, Igo-S64A and Igo1-S64D were purified from bacteria and equal amount of protein was incubated with yeast cell expressing Cdc55-TAP. The proteins bound to the beads were run on 10% SDS-PAGE and probed with anti-TAP antibody. The MBP purified proteins were visualized by coomassie staining of SDS-PAGE gels. WCE denotes whole cell extracts. B) GST-Rim15 and GST-Rim15-kd were purified from yeast cells. Equal amounts of MBP fused Igo1, Igo1-S64A and Igo1-S64D were subjected to in vitro phosphorylation using GST-Rim15 and GST-Rim15-kd respectively. MBP fused proteins were then pulled down with amylose beads and mixed with soluble protein extracts from a yeast strain expressing Cdc55-TAP. The proteins bound to the beads were analysed by western blotting using an anti-TAP antibody. Purified MBP-tagged proteins were visualized by coomassie staining of SDS-PAGE gels. Phosphorylation of Igo1 by Rim15 at S-64 was assayed using a phospho-specific antibody. WCE denotes whole cell extracts. C) TAP eluates from *CDC55*-TAP and untagged strains were analysed by silver staining. D) Phosphatase activity of TAP eluates from *CDC55*-TAP and untagged strains was measured using a colorimetric assay (Millipore). E) Phospho-mimetic mutant of Igo1 (Igo1S64D) inhibits the phosphatase activity of PP2A^Cdc55^. Purified Cdc55 was incubated with equal amount (25 µg) of MBP-Igo1, MBP-Igo1-S64A and MBP-Igo1-S64D respectively. MBP was used as a control. The mixture was then incubated with 500 µM phosphopeptide (Millipore). The release of free phosphate was measured by colorimetric assay (Millipore).

To test whether phosphorylation of Igo1 at S64 results in increased association with PP2A^Cdc55^, we performed an *in vitro* binding assay. We purified wild type Igo1, Igo1-S64A and Igo1-S64D from bacterial cells by attaching a Maltose Binding Peptide (MBP) to their N-termini. We then incubated endosulfine (and its variants) bound to amylose resin *via* the MBP with yeast extracts containing Cdc55-TAP (Tandem Affinity Purification). Specifically Igo1-S64D but not WT Igo1/Igo1-S64A physically interacted with Cdc55 *in vitro* (Figure 3A). We then tested whether phosphorylation of wild type endosulfine by Rim15 results in increased association with Cdc55. For this, we purified either wild type or a Kinase-Dead (kd) version of Rim15 (Rim15-C115A) from yeast cells using a GST affinity tag. We then incubated either wild type or Igo1-S64A or Igo1-S64D bound to amylose resin with Rim15/Rim15-kd for 45 minutes in the presence of ATP. The phospho-mimetic mutant Igo1-S64D interacted with Cdc55 regardless of whether it was incubated with Rim15/Rim15-kd. Wild type Igo1, but not Igo1-S64A, incubated with catalytically active Rim15 interacted with Cdc55 (Figure 3B). These results indicate that phosphorylation of Igo1 at S64 promote its association with PP2A^Cdc55^. We confirmed the phosphorylation of Igo1 at S-64 by Rim15 using a phospho-specific antibody directed against S-64-P which recognized Igo1 incubated with wild-type but not a catalytically dead version of Rim15 (Figure 3B).

To test whether phosphorylation of endosulfine at S64 converts it into an inhibitor of PP2A^Cdc55^, we measured the phosphatase activity of PP2A^Cdc55^ in the presence of Igo1, Igo1-S64A and Igo1-S64D proteins. We purified PP2A^Cdc55^ by attaching a TAP (Tandem Affinity Purification) tag to the C-terminus of Cdc55 (Figure 3C). At first, we measured the phosphatase activity of purified Cdc55 using a phosphorylated peptide as a substrate. Crucially, the TAP eluates from Cdc55-TAP tagged strain but not from an untagged strain, had phosphatase activity (Figure 3D). We then purified MBP-fused versions of Igo1/Igo1-S64D/Igo1-S64A from bacteria and tested their effect on PP2A^Cdc55^ phosphatase activity. Only Igo1-S64D but not Igo1/Igo1-S64A inhibited the phosphatase activity of PP2A^Cdc55^ (Figure 3E). Purified endosulfines had little or no phosphatase activity on their own (Figure S5). These results show that phosphorylation of endosulfine Igo1 at S64 by Rim15 converts it into an inhibitor of PP2A^Cdc55^.

### *dhh1Δ* and *ccr4Δ* do not suppress the G_0_ - and gametogenesis- entry defects of *igo1Δ igo2Δ* cells

**Figure 4:**
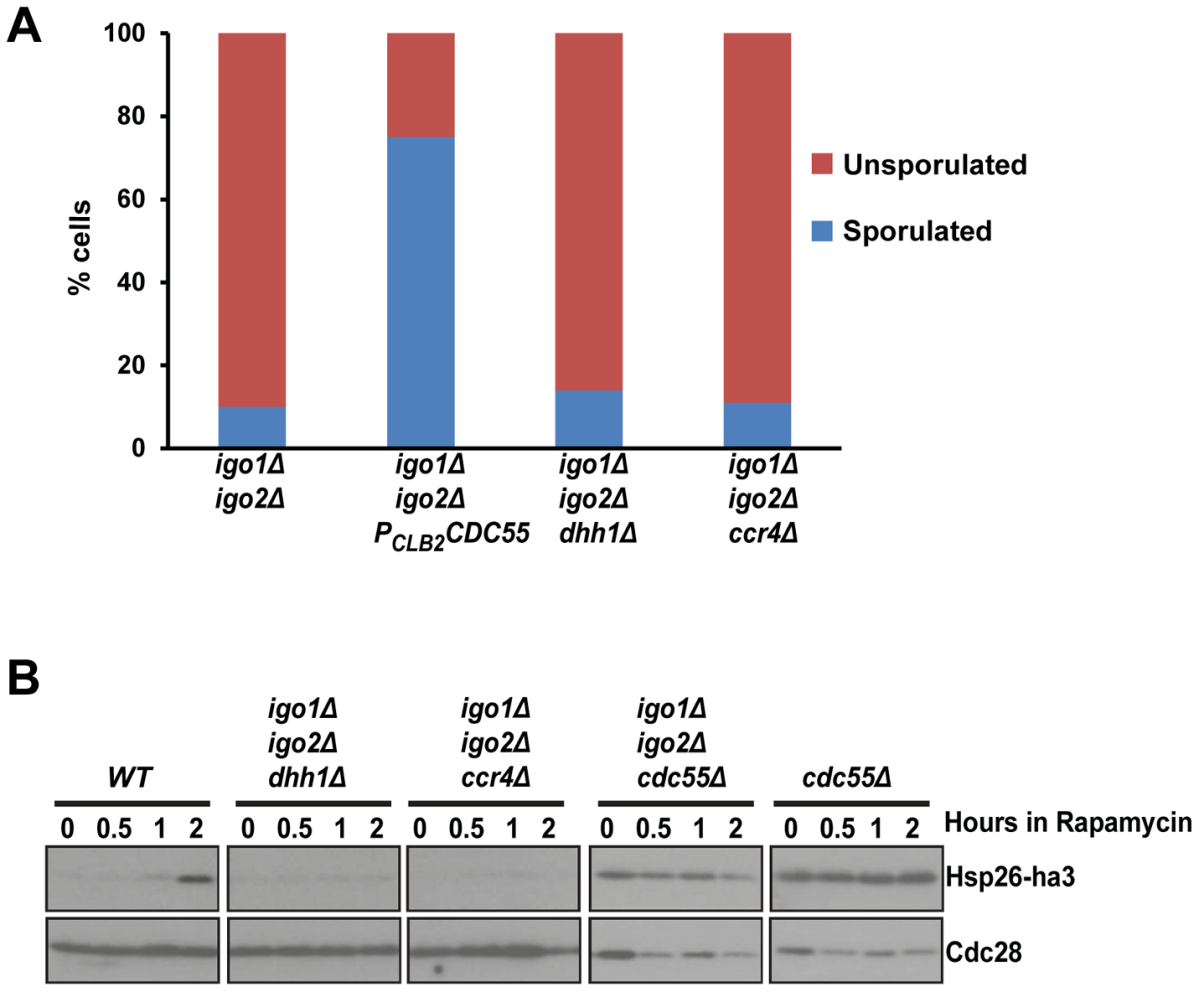
*dhh1Δ* and *ccr4Δ* do not suppress the G_0_ - and gametogenesis- entry defects of endosulfine mutant cells. A) Sporulation efficiency of *igo1Δ igo2Δ*, *igo1Δ igo2Δ P_CLB2_CDC55*, *igo1Δ igo2Δ dhh1Δ* and *igo1Δ igo2Δ ccr4Δ* cells was measured (n = 200). B) Wild type, *igo1Δ igo2Δ*, *cdc55Δ*, *igo1Δ igo2Δ cdc55Δ*, *igo1Δ igo2Δ dhh1Δ* and *igo1Δ igo2Δ ccr4Δ* cells expressing *HSP26*-*ha3* were grown to log phase at 30°C. Rapamycin (final concentration 200 ng/ml) was added to the culture and processed as described above. Whole cell extracts were subjected to SDS-PAGE followed by western analysis using anti-HA and anti-Cdc28 antibodies.

Endosulfines activated by Rim15 were proposed to protect mRNA involved in stress response from the 5′ to 3′ mRNA decay pathway by direct inhibition of decapping enzyme Dhh1 [8]. Consistent with this possibility, deletion of genes *DHH1* and *CCR4*, which are required for 5′ to 3′ decay were reported to suppress entry into quiescence defect of *igo1Δ igo2Δ* cells [8]. We therefore tested whether *dhh1Δ* and *ccr4Δ* also suppress the sporulation defect of *igo1Δ igo2Δ* cells. While *P_CLB2_CDC55* suppressed the sporulation defect of *igo1Δ igo2Δ* cells, both *dhh1Δ* and *ccr4Δ* did not (Figure 4A). We also found that *dhh1Δ* and *ccr4Δ* did not suppress the G_0_ entry defect of *igo1Δ igo2Δ* cells (Figure 4B) contrary to what was previously reported [8]. We do not know the reason for this discrepancy but differences in the strain background used (BY4741 vs. SK1) for the experiments could be an explanation. However, our data are consistent with a simple model which posits that endosulfines regulate entry into quiescence and gametogenesis only through inhibition of PP2A^Cdc55^.

### The Rim15-Endosulfine-PP2A^Cdc55^ pathway regulates entry into gametogenesis independent of G_0_ transcription factors Msn2, Msn4 and Gis1

**Figure 5:**
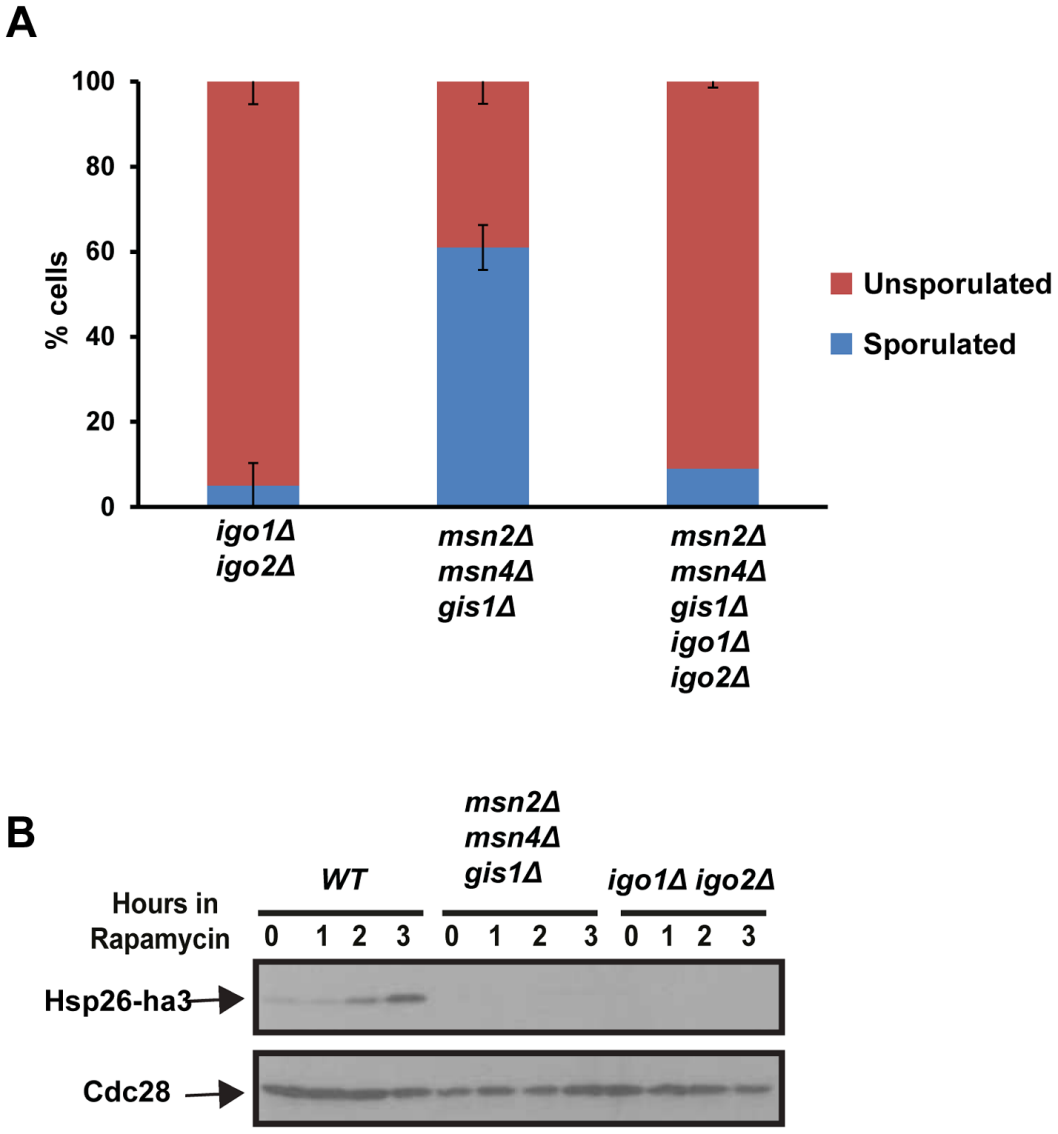
The G_0_-specific transcription factors Msn2, Msn4 and Gis1 are not required for entry into gametogenesis. A) *igo1Δ igo2Δ*, *msn2Δ msn4Δ gis1Δ*, *igo1Δ igo2Δ msn2Δ msn4Δ gis1Δ* mutant cells were incubated on sporulation plates for 24 hours and sporulation efficiency was measured (n = 200). B) *Wild type*, *igo1Δ igo2Δ* and *msn2Δ msn4Δ gis1Δ* cells carrying pRS316-*HSP26*-*ha3* plasmid were grown to log phase. Rapamycin (200 ng/ml) was added to the culture and total protein extract was prepared from cells at the indicated time points and Western analysis was performed using anti-HA and anti-Cdc28 antibodies.

Entry into quiescence is mediated by activation of three master transcription factors namely Msn2, Msn4 and Gis1 [5]. If the roles of Rim15-Endosulfine-PP2A^Cdc55^ module during entries into quiescence and gametogenesis were identical then one would predict that cells lacking the three G_0_–specific transcription factors to be also defective in entry into gametogenesis. However we found that *msn2Δ msn4Δ gis1Δ* cells formed spores (60%) although with defective spore walls (Figure 5A and data not shown). To confirm that the tetrad formation in *msn2Δ msn4Δ gis1Δ* cells was dependent on endosulfines, we deleted *IGO1* and *IGO2* in *msn2Δ msn4Δ gis1Δ* cells. The quintuple mutant cells were defective in forming tetrads like *igo1Δ igo2Δ* cells (Figure 5A). We confirmed that *msn2Δ msn4Δ gis1Δ* cells were defective in entry into quiescence (Figure 5B). These results indicate that Rim15-Endosulfine-PP2A^Cdc55^ pathway regulates entry into gametogenesis independently of activation of G_0_ transcription factors.

### Endosulfines are required for transcriptional induction of *IME1* caused by transfer of cells to sporulation medium

**Figure 6:**
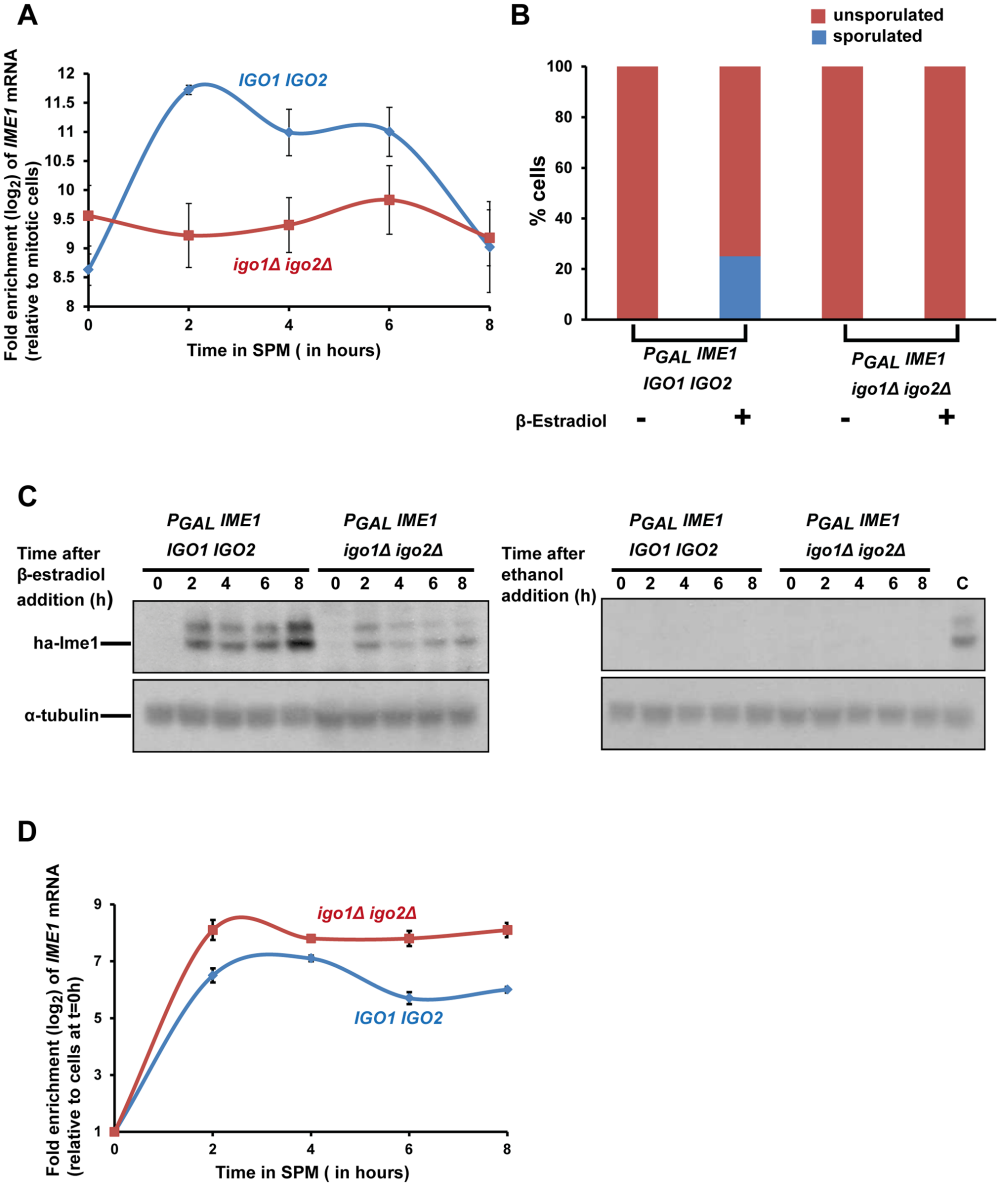
Endosulfines are required for transcriptional induction of *IME1* caused by transfer of cells to sporulation medium. A) Wild-type and *igo1Δ igo2Δ* cells were induced to enter meiosis by transferring them to sporulation medium (SPM). Total RNA was prepared from cells after 0, 2, 4, 6 and 8 hours following transfer to SPM and *IME1* transcript levels were assayed by quantitative RT-PCR. The *IME1* transcript levels were normalized with respect to *ACT1* mRNA and expressed relative to normalized *IME1* transcript levels in mitotically grown cells. B) Wild-type and *igo1Δ igo2Δ* cells containing P*_GAL_*-*ha_3-_IME1 GAL4-ER* were induced to sporulate. Either β-estradiol or ethanol was added to the cultures at t = 0 h. After 24 hours, sporulation efficiency was measured (n = 200). C) Aliquots of cells in B were collected after 0, 2, 4, 6 and 8 hours following addition of β-estradiol/ethanol and total cell extracts were prepared. Immunoblotting was performed using an anti-HA antibody and anti-tubulin antibody. The lane labelled C on the western image on the right contained extracts from β-estradiol treated wild type cells (t = 4 hours) and served as a positive control. D) Aliquots of cells in B were collected after 0, 2, 4, 6 and 8 hours following addition of β-estradiol and total RNA from the cells was prepared. *IME1* mRNA levels were quantified by quantitative RT-PCR and normalized with respect to *ACT1* mRNA. In the graph, *IME1* mRNA levels are expressed relative to *IME1* mRNA levels in cells before induction of *IME1* expression (t = 0 h).

Ime1 is a master transcription factor for expression of early meiotic genes [9]. As indicated above (Figure 1D) Ime1 was not strongly expressed in *igo1Δ igo2Δ* cells after transfer to SPM. In wild type cells, *IME1* is not expressed in glucose-containing nutrient medium but is transcribed at low levels in pre-sporulation medium (which contains acetate as a carbon source) and induced further following transfer to SPM [26]–[28]. We tested whether endosulfines are required for this transcriptional induction of *IME1* by assaying *IME1* mRNA levels by quantitative RT-PCR. In wild type and *igo1Δ igo2Δ* cells grown in pre-sporulation medium (which contains acetate as the carbon source), the levels of *IME1* transcript were around 500-fold higher than in log-phase cells grown in glucose-containing nutrient medium (Figure 6A). However upon transfer to SPM, the *IME1* mRNA levels increased further by about 8-fold after 2 hours in wild type but not in *igo1Δ igo2Δ* cells (Figure 6A). This suggests that endosulfines are required for transcriptional induction of *IME1* caused by transfer to SPM.

### Expression of Ime1 is not sufficient for suppressing the sporulation defect of endosulfine mutants

If the only role of endosulfines in entry into gametogenesis was to activate transcription of *IME1*, then ectopic expression of *IME1* should bypass the sporulation defect of *igo1Δ igo2Δ* cells. To test this, we constructed wild type and *igo1Δ igo2Δ* strains in which *IME1* expression can be induced by addition of β-estradiol to the medium using the *P_GAL_*/Gal4-ER system [29]. We transferred wild type and *igo1Δ igo2Δ* cells to SPM in the presence or absence of β-estradiol. While wild type cells sporulated in the presence of β-estradiol, *igo1Δ igo2Δ* cells failed to do so (Figure 6B). Ime1 was expressed in β-estradiol treated *igo1Δ igo2Δ* cells although at a lower level compared to wild type cells (Figure 6C). Since endosulfines have been implicated in mRNA stability [8], we tested whether the difference in the Ime1 levels in the two strains was due to difference in the *IME1* transcript levels. Quantitative RT-PCR analyses revealed that the *IME1* transcript levels were induced to similar extent in wild type and *igo1Δ igo2Δ* strains and remained relatively unchanged up to 8 hours following induction (Figure 6D). This suggests that endosulfines are not required for regulating *IME1* mRNA stability. Decreased Ime1 levels in *igo1Δ igo2Δ* cells could be caused by either decreased translational efficiency of *IME1* mRNA or decreased Ime1 stability. These results indicate that endosulfines promote entry into gametogenesis independently of regulating *IME1* expression.

### Endosulfines are required for pre-meiotic autophagy

**Figure 7:**
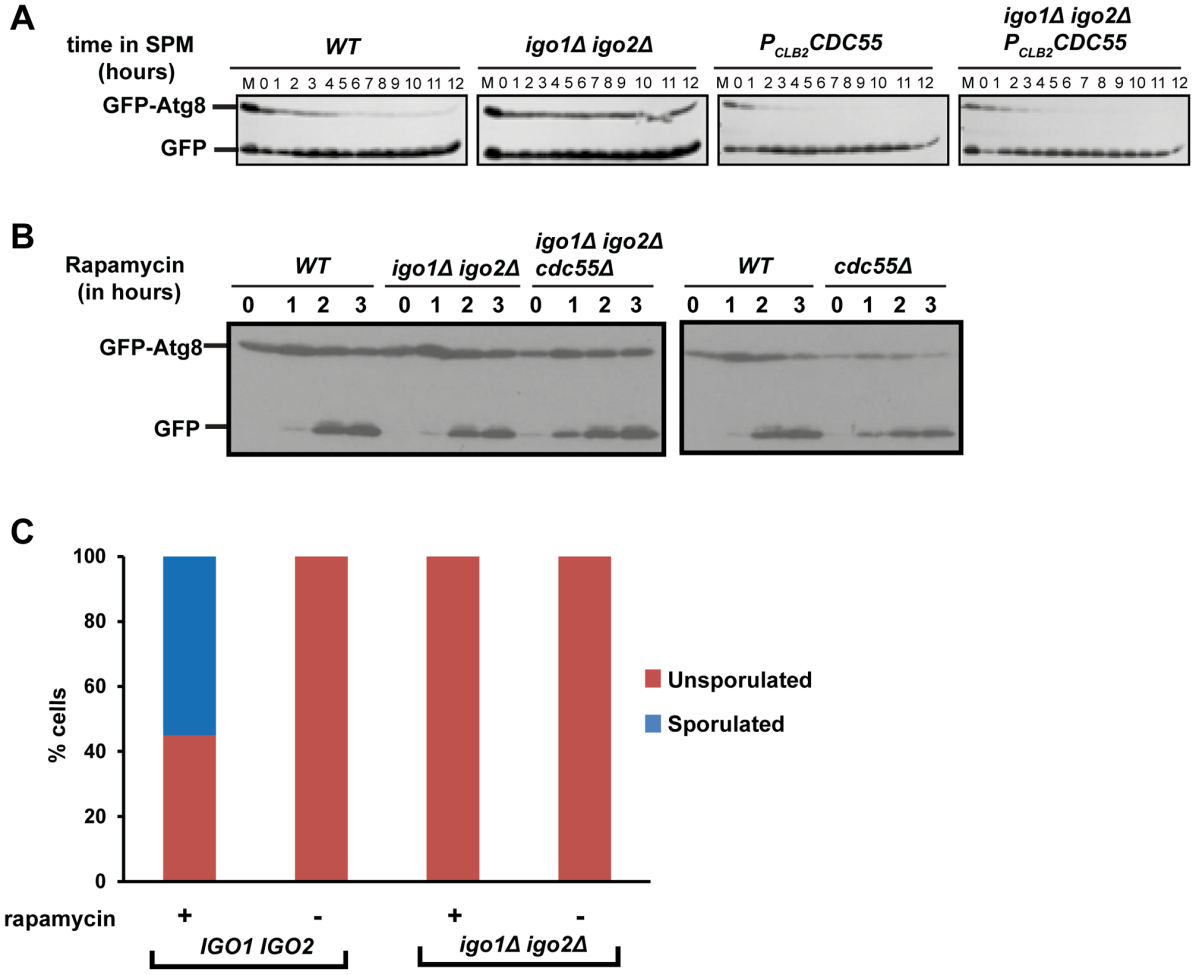
Endosulfine mutant is defective in pre-meiotic autophagy but not for autophagy induced by rapamycin treatment. A) Wild type, *P_CLB2_CDC55*, *igo1Δ igo2Δ* and *igo1Δ igo2Δ P_CLB2_CDC55* cells expressing GFP-Atg8 were induced to sporulation. Samples were collected at indicated times and total cell extract was prepared. Western analysis was performed using anti-GFP antibody. B) Wild type, *cdc55Δ*, *igo1Δ igo2Δ* and *igo1Δ igo2Δ cdc55Δ* cells expressing GFP-Atg8 were grown to log phase in YEPD and rapamycin (final concentration 200 ng/ml) was added to the cultures. The cultures were incubated further for 3 hours. Cells were collected at indicated time points, total protein extract was prepared and immunoblot analysis was performed using anti-GFP antibody. C) Wild-type, *igo1Δ igo2Δ* cells were grown to saturation in YEPD, rapamycin (final concentration 200 ng/µl) was added to the culture and grown for another 24 hours. Sporulation efficiency was measured by counting the total number of monad, dyad and triads/tetrads in the culture (n = 200).

Rim15 is required for autophagy induced by inhibition of PKA and Sch9 but not for autophagy induced by rapamycin treatment [30]. Since autophagy is required for spore formation in yeast [31], we tested whether endosulfines are required for autophagy during entry into gametogenesis. Autophagy can be assayed by following proteolytic cleavage of GFP-Atg8, which is a N-terminal fusion of GFP to Atg8 (a ubiquitin-like protein required for formation of autophagosomal membranes) [32]. We induced wild type, *P_CLB2_CDC55*, *igo1Δ igo2Δ* and *igo1Δ igo2Δ P_CLB2_CDC55* cells to enter meiosis by transferring them to SPM and assayed autophagy. In wild type cells, GFP-Atg8 underwent proteolytic cleavage after 2 hours into SPM (Figure 7A). In contrast, GFP-Atg8 remained intact in *igo1Δ igo2Δ* cells even after 12 hours in SPM (Figure 7A). Strikingly, GFP-Atg8 was cleaved earlier in *P_CLB2_CDC55* and *igo1Δ igo2Δ P_CLB2_CDC55* cells in comparison to wild type cells. These results are consistent with the hypothesis that PP2A^Cdc55^ inhibits pre-meiotic autophagy and that this inhibition is overcome by endosulfines after transfer to SPM.

We then tested whether endosulfines are required for autophagy induced by rapamycin treatment. We treated wild type, *cdc55Δ*, *igo1Δ igo2Δ* and *igo1Δ igo2Δ cdc55Δ* cells with rapamycin and assayed autophagy by western analysis. While autophagy in *cdc55Δ* cells was slightly advanced in comparison to wild type cells, endosulfine mutant cells underwent autophagy as efficiently as wild type cells (Figure 7B). We also found that endosulfines were not required for autophagy triggered by nitrogen starvation (Figure S6). Since rapamycin treatment and nitrogen starvation trigger autophagy by inhibiting TORC1, our results indicate that endosulfines are not required for autophagy induced by inhibition of TORC1 signalling. Rapamycin treatment of diploid cells induces sporulation [33]. While rapamycin–treated wild type cells formed tetrads after 24 hours, *igo1Δ igo2Δ* cells did not (Figure 7C). This suggests that induction of autophagy *per se* is insufficient for rescuing the sporulation defect of endosulfine mutant cells.

To determine the role of autophagy in sporulation, we induced wild type and *atg1Δ* cells (*ATG1* encodes a serine-threonine kinase required for autophagy) to enter meiosis by transferring them to SPM. Wild type cells completed pre-meiotic DNA replication after 4–5 hours and underwent two rounds of nuclear division to form 45% tetrads (Figure S7A–B). Although the kinetics of Rec8 expression in wild type and *atg1Δ* cells were similar (Figure S7B), *atg1Δ* cells were delayed in initiation of pre-meiotic DNA replication by about 1–2 hours in comparison to wild type cells. Expression of Cdc5 (marker for mid-meiosis) in *atg1Δ* cells was delayed by about 3 hours relative to wild type cells (Figure S7C). However *atg1Δ* cells failed to undergo nuclear divisions and remained largely mononucleate with prophase I spindles after 10 hours in SPM (Figure S7B and data not shown). Since the phenotype of *atg1Δ* cells is distinct from that of *igo1Δ igo2Δ* cells (which fail to enter gametogenesis as indicated in Figure 1), we conclude that endosulfines regulate entry into gametogenesis independently of controlling pre-meiotic autophagy.

Ume6 associates the histone deacetylase Sin3/Rpd3 to negatively regulate entry into gametogenesis [11]. Interestingly, Ume6 is phosphorylated during sporulation in a Rim15-dependent manner [34]. If endosulfines promote entry into gametogenesis through inhibition of Ume6 and Sin3/Rpd3, then *ume6Δ* and *rpd3Δ* should suppress *igo1Δ igo2Δ*. However *ume6Δ* and *rpd3Δ* did not suppress the poor sporulation efficiency of *igo1Δ igo2Δ* cells (Figure S8). Surprisingly, both *ume6Δ* and *rpd3Δ* completely abolished the ability of *igo1Δ igo2Δ* cells to form tetrads (Figure S8). This suggests that endosulfines and Ume6/Rpd3 regulate spore formation *via* independent pathways.

## Discussion

**Figure 8:**
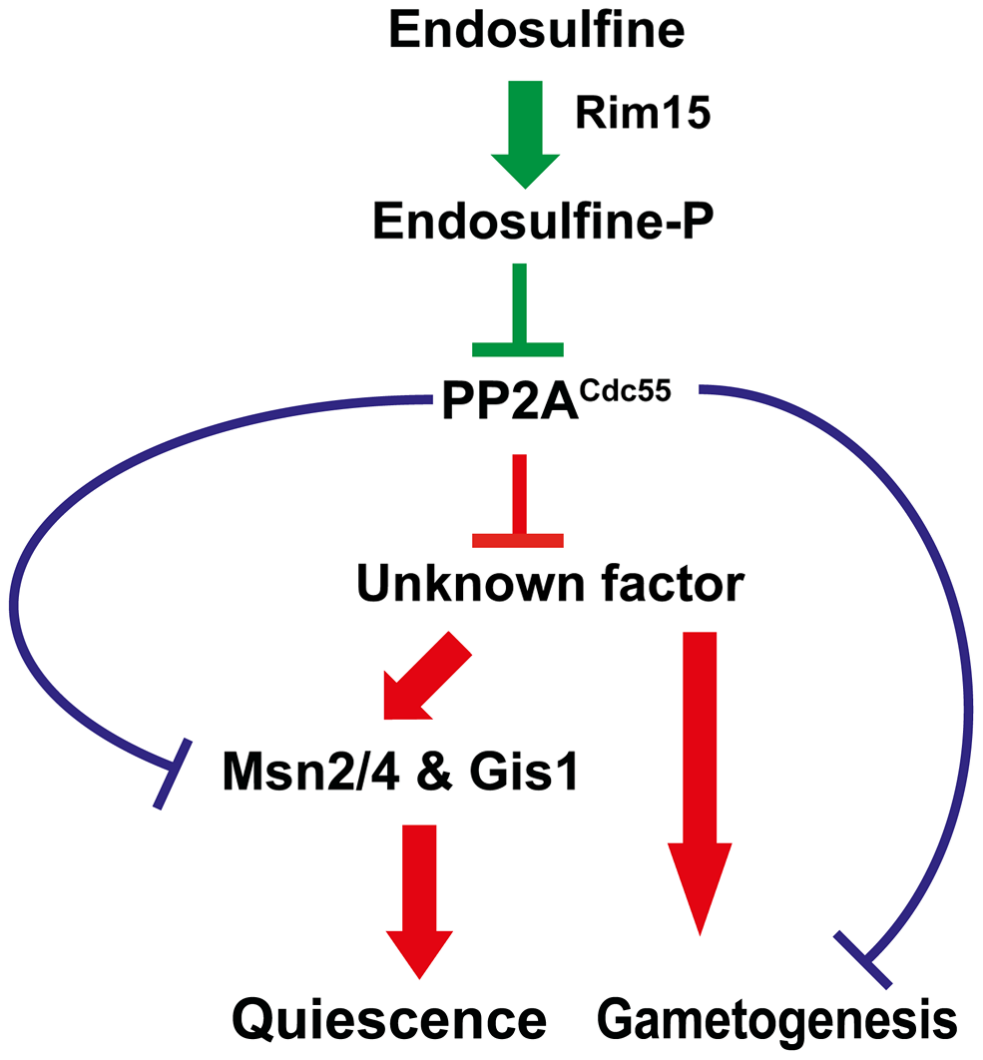
Regulation of entry into gametogenesis and quiescence by the Rim15-Endosulfine-PP2A^Cdc55^ signalling module. Phosphorylation of endosulfine by Rim15 converts it into an inhibitor of PP2A^Cdc55^ and thus leading to entry into gametogenesis and quiescence. Entry into quiescence is driven by activation of transcription factors Msn2, Msn4 and Gis1. PP2A^Cdc55^ might inhibit a positive regulator of entry into gametogenesis and quiescence. Alternatively, PP2A^Cdc55^ could inhibit entry into quiescence and gametogenesis by dephosphorylating distinct substrates.

We have shown that a signalling module consisting of a serine-threonine kinase Rim15, endosulfine Igo1/2 and PP2A^Cdc55^ regulates entry into gametogenesis and quiescence in budding yeast (Figure 8). While our manuscript was in preparation another group reported that Rim15-Endosulfine-PP2A^Cdc55^ pathway is required for onset of quiescence in yeast cells [35]. We show that the signalling module also regulates entry into gametogenesis *via* a mechanism that is independent of entry into quiescence. Remarkably both studies show that Rim15-Endosulfine-PP2A^Cdc55^ signalling module in budding yeast mechanistically works like the Greatwall Kinase-endosulfine-PP2A-B55δ pathway that regulates mitotic entry in Xenopus egg extracts [17], [18].

Entry into quiescence is controlled by activation of the G_0_ transcription factors Msn2, Msn4 and Gis1. We show that entry into gametogenesis is not dependent on the G_0_ transcription factors suggesting that the Rim15-Endosulfine-PP2A^Cdc55^ regulates entry into G_0_ and gametogenesis by distinct mechanisms. Precisely how PP2A^Cdc55^ prevents entry into gametogenesis and G_0_ remains unknown. Although the stress-responsive transcription factor Gis1 is hyperphosphorylated in *cdc55* mutant cells [35], it is not known whether it is a direct substrate of PP2A^Cdc55^. It is possible that PP2A^Cdc55^ inhibits a factor that is a positive regulator of entry into both gametogenesis and G_0_ (Figure 8). Alternatively, PP2A^Cdc55^ might inhibit entry into gametogenesis and G_0_ by dephosphorylation of distinct substrates (Figure 8). Comparing the phosphoproteomes of wild type, *cdc55* and *igo1Δ igo2Δ* cells during entry into gametogenesis and quiescence would be illuminating. Contrary to previous observations [8], we did not find any genetic evidence for endosulfine function in controlling 5′ to 3′ mRNA decay pathway. We suggest that endosulfines regulate entry into both gametogenesis and G_0_ only *via* inhibition of PP2A^Cdc55^ (Figure 8).

We have shown that the Rim15-Endosulfine-PP2A^Cdc55^ is required for pre-meiotic autophagy. However the inability to undergo autophagy does not account for the meiotic phenotype of *igo1Δ igo2Δ* cells as *atg1Δ* cells (defective in autophagy) enter gametogenesis but fail to undergo any nuclear divisions. Induction of autophagy in *igo1Δ igo2Δ* cells by rapamycin treatment did not rescue the sporulation defect. Expression of Ime1, the master transcription factor for early meiotic genes, also did not rescue the sporulation defect of *igo1Δ igo2Δ* cells. These results indicate that the Rim15-Endosulfine-PP2A^Cdc55^ module regulates entry into gametogenesis independently of controlling pre-meiotic autophagy and Ime1 expression. Interestingly Cdc55 has been found to physically interact with Atg1 and Atg18, two proteins required for autophagy, in interactome screens [36], [37]. It will be informative to test whether these interactions are altered during entry into gametogenesis.

Precisely how Rim15/Gwl phosphorylated endosulfine inhibits PP2A^Cdc55^ activity is not known. Structural analyses of PP2A^Cdc55^-endosulfine complex would be illuminating in this respect. It is also important to determine whether endosulfine inhibits PP2A^Cdc55^ activity towards all or only a specific subset of its physiological substrates. Hypomorphic mutations in *CDC55* suppress the dyad phenotype of *spo12Δ* strains (Gary William Kerr and Prakash Arumugam, unpublished observations). This is consistent with antagonistic roles of Spo12 and PP2A^Cdc55^ in FEAR pathway and exit from meiosis I [23], [38], [39]. In contrast to *cdc55* hypomorphic alleles, *igo1-S64D* did not suppress the *spo12Δ* dyad phenotype (data not shown) suggesting that phosphorylated endosulfine inhibits PP2A^Cdc55^ activity towards only some of its cellular substrates.

Testing whether endosulfines are required for quiescence and gametogenesis in mammalian cells would be very interesting. Notably, expression of endosulfines was first noted in brains [40] and was decreased several fold in patients with neurodegenerative diseases [41]. Given the high conservation of this signalling module, deconstructing its mechanism in budding yeast might give insights into regulation of mitosis in human cells, and *vice versa*.

## Materials and Methods

### Yeast strains and plasmids

A complete list of yeast strains and their genotypes can be found in Table S1.

### Purification of Igo1 and Rim15

The MBP fused wild-type and mutant forms (S64A and S64D respectively) of Igo1 were expressed and purified from bacteria using the amylose resin (NEB) according to the manufacturer's instructions. Briefly, *E. coli* cells expressing the MBP fusion proteins were grown overnight and sub-cultured in 2× TY medium containing 0.2% glucose and grown at 37°C to an OD_600 nm_ of ∼0.5. IPTG (Isopropyl β-D-1-thiogalactopyranoside) was added to the culture to a final concentration of 0.2 mM and cells were allowed to grow for another 3 hours at 37°C. Cells were harvested at 4000 rpm for 10 minutes at room temperature and resuspended in 5 ml of buffer A (20 mM Tris-Cl, pH 7.5, 250 mM NaCl, 1 mM EDTA, 5 mM β-mercaptoethanol, Roche Complete EDTA-free Protease Inhibitors and 100 mM PMSF) and stored at −20°C after freezing it in liquid N_2_. Cells were thawed in cold water and lysed by sonication (40 Amp, 5×15 seconds, 1–2 minutes interval between each pulse). Cells were centrifuged at 13,200 rpm for 20 minutes at 4°C and supernatant was transferred to separate tubes. Total amount of protein was measured using the Bradford assay [42]. Equal amount of 50% slurry of Amylose resin (pre-equilibrated in buffer A) was added to the cell lysate. The mixture was incubated for 20 minutes on ice. Beads were collected at low speed (2000 rpm, 1 min, 4°C), washed thrice with 1 ml of buffer A. Proteins bound to beads were recovered by elution with maltose (10 mM) or by adding 2× SDS sample buffer to the beads followed by incubation at 95°C for 5 minutes.

GST-tagged wild-type or mutant forms of Rim15 was purified from yeast cells. Briefly, cells carrying the plasmids (encoding either wild-type or mutant Rim15) were grown to log phase in SD –URA medium at 30°C containing 2% raffinose. After allowing the cultures to reach an OD_600 nm_ ∼1.0, YPG (1% Yeast extract, 2% bactopeptone and 2% galactose) was added to the culture and grown for another 4 hours at 30°C. Cells were collected, washed in cold water and frozen in liquid N_2_ and stored at −80°C. Cells were thawed , resuspended in lysis buffer (50 mM Tris-Cl, pH 7.5, 100 mM NaCl, 1 mM EDTA, 1% NP-40, 1 mM PMSF and Roche Complete EDTA-free Protease Inhibitors) and lysed by using glass beads. Total amount of protein was measured; equal amount of protein was mixed with 150 µl of 50% slurry of GST beads (pre-equilibrated in lysis buffer) and rotated at 4°C for 1 hour. Beads were collected and washed once with lysis buffer, twice with lysis buffer +250 mM NaCl and twice with lysis buffer +500 mM NaCl. The GST-fused proteins were eluted using 10 mM reduced glutathione.

### *In vitro* interaction between Igo1 and Cdc55

Yeast cells expressing Cdc55-TAP were grown to log phase at 30°C in YEPD medium, harvested at 4000 rpm for 5 minutes at 4°C. The cell pellets were stored at −80°C after freezing it in liquid N_2_. The pellet was thawed, resuspended in yeast lysis buffer (50 mM Tris-Cl, pH 7.5, 100 mM NaCl, 1 mM EDTA, 1% NP-40, 1 mM PMSF and Roche Complete EDTA-free Protease Inhibitors) and lysed by using glass beads. Protein concentration was measured by Bradford method. MBP fused wild-type and mutant Igo1 proteins were purified as described above. Equal amounts of bead bound proteins were added to equal amounts of total yeast cell extract. The mixture was incubated on ice for 20 minutes. The beads were collected by centrifugation, washed three times with lysis buffer, resuspended in SDS sample buffer, boiled and run on 10% SDS-PAGE.

GST fused Rim15 and Rim15-kd proteins were purified as described above and the purified protein was used to phosphorylate purified MBP-fused Igo1. The reaction was carried out in kinase buffer (50 mM Tris-Cl, pH7.5, 20 mM MgCl_2_, 1 mM DTT) containing 1 mM ATP at room temperature for 45 minutes. Beads were collected, mixed with equal amount of yeast cell extract containing Cdc55-TAP and incubated on ice for 20 minutes. The beads were washed three times with lysis buffer, resuspended in 2× SDS sample buffer, boiled and analyzed by Western blotting following SDS-PAGE.

### Phosphatase assay

Phosphatase assay was carried out using the Ser/Thr phosphatase assay kit containing a phospho-peptide as a substrate (from Millipore). Briefly, strain expressing TAP-tagged Cdc55 was grown in 1 litre of YEPD medium to log-phase. Cells were harvested, resuspended in 5 ml of yeast lysis buffer and soluble extracts were prepared by bead beating. The extract was mixed with 0.2 ml of IgG sepharose beads (pre-equilibrated in lysis buffer) and the mixture was incubated for 2 hours on a rotary wheel at 4°C. The beads were precipitated, washed 4 times with lysis buffer, once with TEV cleavage buffer (10 mM Tris-Cl, pH 7.5, 150 mM NaCl, 0.5 mM EDTA, 0.1% NP-40 and 1 mM DTT) and resuspended in 350 µl of TEV cleavage buffer. The bound protein was cleaved and eluted from the beads after incubating overnight at 4°C with 15 U of TEV protease (Invitrogen). The eluted protein was then used for phosphatase assay. Purified Cdc55 was mixed with equal amount (25 µg) of MBP-fused Igo1 or Igo1-S64A or Igo1-S64D or MBP alone and incubated for 20 minutes on ice. The mixture was then incubated with 500 µM of phospho-peptide for 1 hour at 30°C. The reaction was terminated by addition of malachite green solution provided with the kit and absorbance was measured at 620 nm.

### *In situ* immunofluorescence

For *in situs*, cells from 1 ml of yeast culture were fixed for 15 minutes with 3.7% formaldehyde, pelleted and resuspended in 100 mM K-phosphate buffer (pH 6.4) containing 3.7% formaldehyde and kept overnight on ice. Immunostaining was performed as previously described [23]. The following primary antibodies were used: monoclonal rat anti-α-tubulin 1∶500 (Serotec), monoclonal mouse anti-HA 1∶500 (Covance). Secondary antibodies, pre-absorbed against sera from other species used in labeling, were conjugated with Cy3 or Cy5 (Chemicon) and diluted 1∶500 (Cy3) or 1∶50 (Cy5). DNA was visualized by staining with DAPI.

### Microscopy

Images were acquired using a Nikon TE-2000 inverted microscope with a 100×1.49 N.A. objective lens equipped with a Photometrics Coolsnap-HQ2 liquid cooled CCD camera (Photometrics, Tucson, AZ). 16 Z-stacks (spacing = 0.2 µm) Exposure times of 1 second were used for both Cy3 and Cy5, and 0.25 seconds for DAPI. Images were analysed using Metamorph (version 7.5.2.0 MAG Biosystems Software).

### Immunoblotting

Whole cell extracts were prepared by cell breakage with glass beads in 20% Trichloroacetic acid. Cell pellets were resuspended in 2× SDS sample buffer, neutralized with 2M Tris base and proteins were denatured by heating the samples at 95°C for 5′. After centrifugation, protein samples were electrophoresed on 10% SDS-PAGE gels. The HA epitope was detected by mouse monoclonal antibody 16B12 at 1∶1000. Goat anti-Cdc5 (Santa Cruz SC-6733) antibody, Goat anti-Cdc28 (Santa Cruz-6708) antibody, mouse anti-Pk (Serotec) antibody, mouse anti-GFP (Roche) antibody and rabbit anti-TAP antibody (Pierce) were all used at 1∶1000 dilution. Myc epitope was detected using the 9E10 antibody (Cambridge Biosciences) at 1∶1000 dilution. Phospho-specific antibody was raised against the phosphorylated synthetic peptide KRKYFDpSGDYALC (pS indicates phosphoserine) by Eurogentec.

For phos-tag gels, TCA extracts were prepared as above and analysed as previously described [25] with a few modifications. Briefly 12.5% polyacrylamide gels were prepared and Phos-Tag (Wako) was added at its final concentration of 50 µM to the separating gel mixture before polymerization. Electrophoresis was performed at a constant current of 30 mA at room temp. After electrophoresis, gels were first soaked in Transfer Buffer (25 mM Tris, 192 mM Glycine, 10% methanol) containing 1 mM EDTA for 20 minutes (2×10 minutes) and then in Transfer Buffer for 30 minutes (3×10 minutes). Electrotransfer onto PVDF membrane was done at a constant voltage of 36 V for 16 hours at 4°C.

### Silver staining

After running the protein sample on 10% SDS-PAGE, the gel was washed once with water and then fixed with 100 ml of fixative (50% methanol and 5% acetic acid) for 2 hours. The gel was washed once with 100 ml of 20% ethanol and twice with water. The gel was sensitized with 100 ml of 0.02% sodium thiosulfate for 1 minute and washed immediately with water. The gel was incubated with 100 ml of silver nitrate (0.1% in water) solution containing 20 µl of 37% formaldehyde and kept for 20 minutes at 4°C in dark. The gel was then washed again with water and 100 ml of developing solution (2.5% sodium carbonate 0.0185% formaldehyde) was added. After the bands were visible, 5% acetic acid was added to terminate the reaction.

### Analysis of mRNA by quantitative RT-PCR

Total RNA was extracted from yeast cell pellets using the MasterPure Yeast RNA purification kit (Epicentre). RNA integrity was confirmed by agarose gel electrophoretic analysis after denaturation with formamide. Reverse transcription reactions were performed on 0.5 µg of DNAase I-treated RNA with Oligo-dT, using the GoScript Reverse Transcription System (Promega). Quantitative real-time PCR primers for analyzing *IME1* and *ACT1* were designed as previously described [43] and their specificity was confirmed by melt curve analyses. cDNA reactions were diluted 100-fold, and triplicate quantitative real-time PCRs were performed in a Rotor-Gene Q (Qiagen) using the 2× Rotor-Gene SYBR Green PCR kit (Qiagen). Reactions were analyzed using RotorGene Q software by the comparative *C_T_* method, normalizing *IME1* mRNA levels against the *ACT1* reference gene.

### Other techniques

Induction of sporulation was carried out as previously described [44]. To measure sporulation efficiency of yeast strains on solid media, cells were streak purified on YEPD plates. Three single colonies were patched onto YEPD plates. After 24 h of growth at 30°C, cells were patched onto Sporulation plates (0.82% Sodium acetate, 0.19% Potassium chloride, 0.035% Magnesium sulphate, 0.12% Sodium chloride and 1.5% Agar) and incubated at 30°C for 24 h. Sporulation efficiency was assayed using a light microscope. To induce *GAL1-IME1* expression, β-estradiol was added to the cultures at the final concentration of 1 µM. The DNA content of sporulating cells was measured by flow cytometry as previously described [45].

## Supporting Information

Figure S1 The sporulation defect of endosulfine mutant cells is not due to their failure to exit from stationary phase. Wild-type and *igo1Δ igo2Δ* cells were grown to mid-log phase in YEPA medium. Cells were then transferred to sporulation medium (SPM). A) DNA content was measured by flow cytometry over a period of 24 hours. B) Spore formation in the two strains after 24 hours was assayed by light microscopy.
(PDF)

Figure S2 The effect of phospho-mimetic mutation *igo1-S64D* on sporulation efficiency is independent of Rim15 function. *rim15Δ* cells and *igo1Δ igo2Δ* cells containing either pRS303-*IGO1-myc8* or pRS303-*IGO1-S64A-myc8* or pRS303-*IGO1-S64D-myc8* or *rim15Δ* pRS303-*IGO1-S64D-myc8* were incubated for 24 hours on sporulation plates and percentage of sporulated cells were counted using a light microscope. Values are expressed as mean ± s.e.m of 3 independent measurements.
(PDF)

Figure S3 The endosulfine Igo1 is phosphorylated at S-64 during entry into gametogenesis. A) Strains expressing either Igo1-myc8 or Igo1S64A-myc8 cells induced to sporulate. Cells were collected at indicated time points and TCA extracts were prepared. Protein samples were loaded on phos-tag or normal SDS-PAGE gels and analysed by western blotting using anti-Myc antibody. B) DNA content in the two cultures was measured by flow cytometry over a period of 8 hours.
(PDF)

Figure S4 The conserved PKA site in Igo1 is dispensable for entry into gametogenesis. A) Conserved PKA site at the C-termini of budding yeast endosulfines Igo1, Igo2 and human endosulfines ENSA and ARPP-19. B) Wild type, *igo1Δ igo2Δ*, igo1*-S105D igo2Δ* and *igo1-S105A igo2Δ* cells were incubated on sporulation plates for 24 hours and the number of sporulated (includes monad, dyad, Tri-/terads) and unsporulated cells were counted using a light microscope.
(PDF)

Figure S5 Purified endosulfine has no phosphatase activity. 25 µg of purified MBP, MBP-Igo1, MBP-Igo1S64A and MBP-Igo1S64D was incubated with 500 µM phosphopeptide (Millipore). The release of free phosphate was measured using a colorimetric assay (Millipore). TAP eluates from *CDC55-TAP* and untagged strains were used as positive and negative controls respectively for the phosphatase assay.
(PDF)

Figure S6 Endosulfines are not required for autophagy induced by nitrogen starvation. Wild type, *cdc55Δ*, *igo1Δ igo2Δ* and *igo1Δ igo2Δ cdc55Δ* cells expressing GFP-Atg8 were grown to log phase in YEPD and then transferred to nitrogen deprivation medium. The cultures were incubated further for 3 hours. Cells were collected at indicated time points, total protein extract was prepared and immunoblot analysis was performed using anti-GFP antibody.
(PDF)

Figure S7 The *atg1Δ* strains enter gametogenesis but fail to undergo any meiotic nuclear divisions. Wild-type and *atg1Δ* cells were induced to enter meiosis by transferring them to SPM. A) Pre-meiotic DNA replication in the cultures was assayed by flow cytometry. B) Kinetics of nuclear division of cells was measured after staining cells with DAPI (n = 100). Rec8 expression was monitored by in situ immunofluorescence using an anti-HA antibody. C) Whole-cell extracts from meiotic cultures taken every hour from 0–10 hours was prepared by TCA method. Protein samples were run on 10% SDS-PAGE, transferred to nitrocellulose membrane and probed with anti-Cdc5 and Cdc28 antibody respectively.
(PDF)

Figure S8 *ume6Δ* and *rpd3Δ* do not suppress the sporulation defect of *igo1Δ igo2Δ* cells. Wild-type, *ume6Δ*, *rpd3Δ*, *igo1Δ igo2Δ*, *igo1Δ igo2Δ ume6Δ* and *igo1Δ igo2Δ rpd3Δ* cells were incubated for 24 hours on sporulation plates and number of sporulated (includes monad, dyad, Tri-/tetrads) and unsporulated cells were counted using a light microscope. The experiment was repeated 3 times and 200 cells were counted every time for each strain.
(PDF)

Table S1 List of yeast strains used. All yeast strains are derivatives of SK1 and have the following markers, unless otherwise stated. *ho::LYS2/ho::LYS2, ura3/ura3, leu2::hisG/leu2::hisG, trp1::hisG/trp1::hisG, his3::hisG/his3::hisG, lys2/lys2.*
(PDF)
