# Supplementary figures and images for: The Rim15-Endosulfine-PP2ACdc55 Signalling Module Regulates Entry into Gametogenesis and Quiescence via Distinct Mechanisms in Budding Yeast
Source: PLoS Genet. 2014 Jun 26;10(6):e1004456. doi: 10.1371/journal.pgen.1004456 (PMC4072559; doi:10.1371/journal.pgen.1004456)

**A**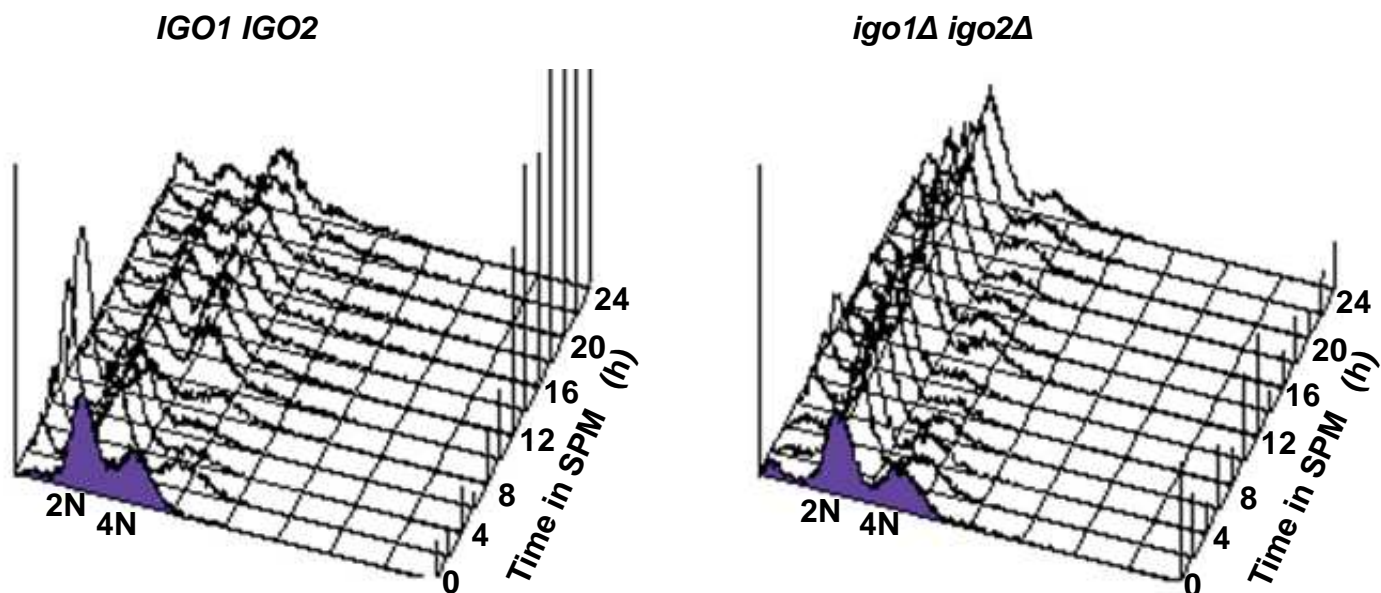**B**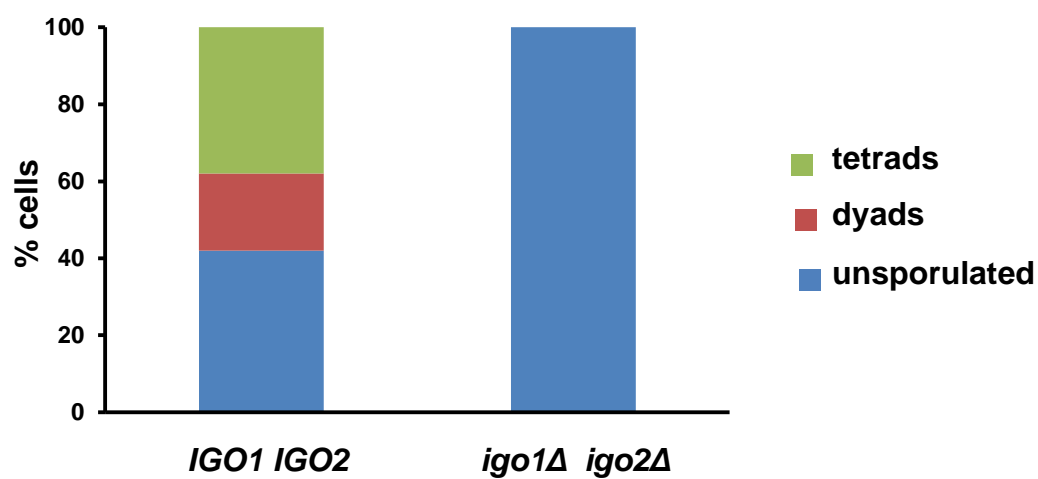**Figure S1**

Supplement: Figure S1 — The sporulation defect of endosulfine mutant cells is not due to their failure to exit from stationary phase. Wild-type and igo1Δ igo2Δ cells were grown to mid-log phase in YEPA medium. Cells were then transferred to sporulation medium (SPM). A) DNA content was measured by flow cytometry over a period of 24 hours. B) Spore formation in the two strains after 24 hours was assayed by light microscopy. (PDF) [file pgen.1004456.s001.pdf]

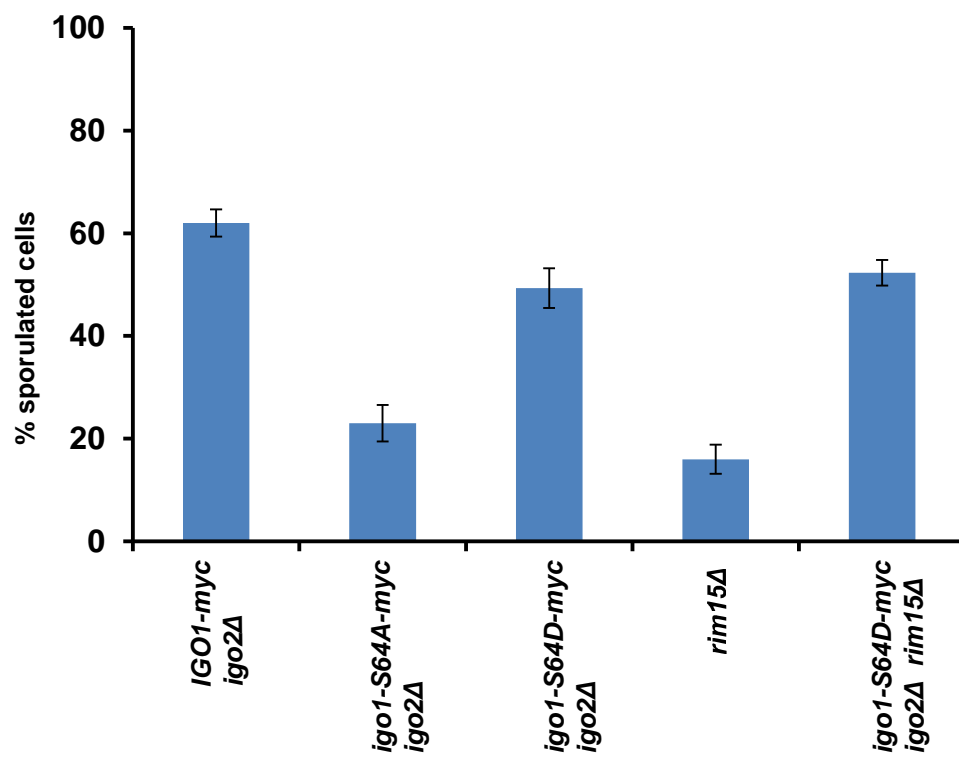

**Figure S2**

Supplement: Figure S2 — The effect of phospho-mimetic mutation igo1-S64D on sporulation efficiency is independent of Rim15 function. rim15Δ cells and igo1Δ igo2Δ cells containing either pRS303-IGO1-myc8 or pRS303-IGO1-S64A-myc8 or pRS303-IGO1-S64D-myc8 or rim15Δ pRS303-IGO1-S64D-myc8 were incubated for 24 hours on sporulation plates and percentage of sporulated cells were counted using a light microscope. Values are expressed as mean ± s.e.m of 3 independent measurements. (PDF) [file pgen.1004456.s002.pdf]

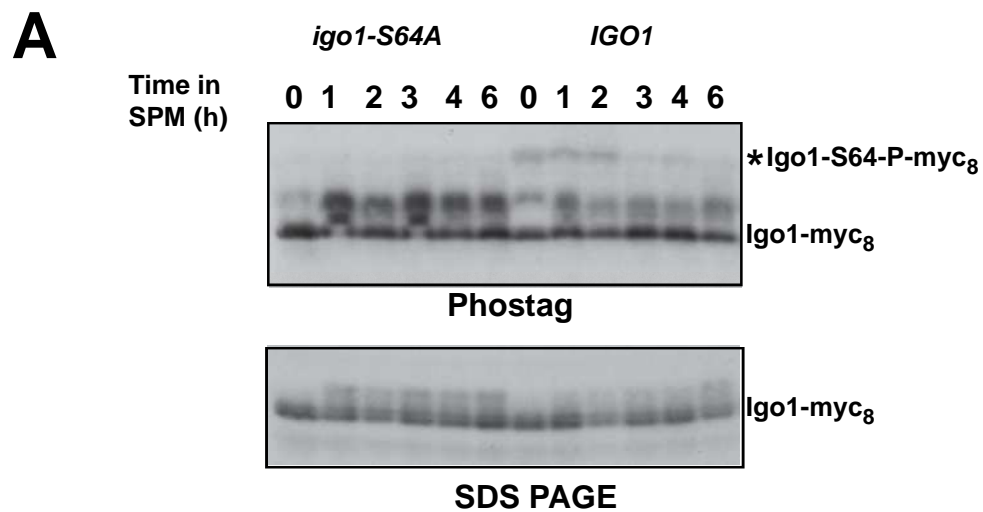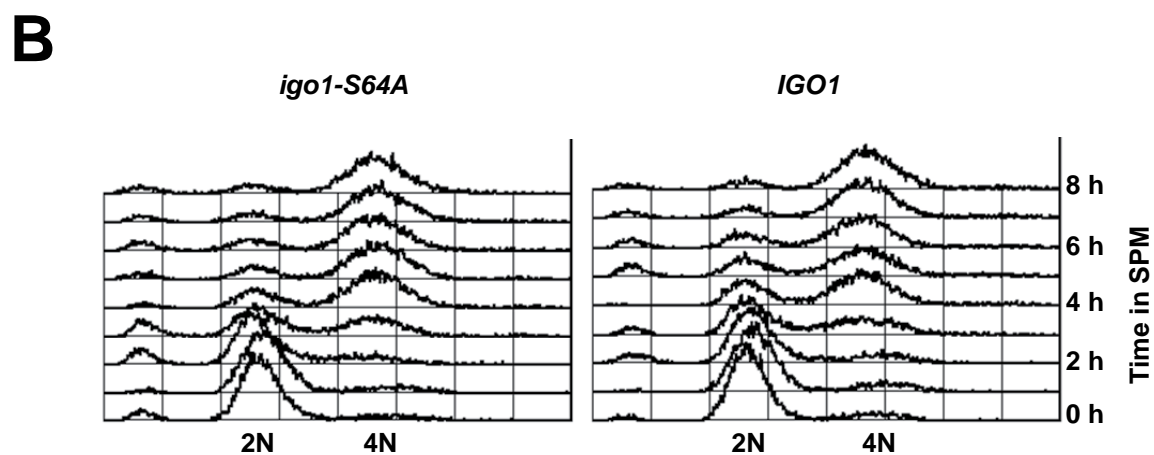

**Figure S3**

Supplement: Figure S3 — The endosulfine Igo1 is phosphorylated at S-64 during entry into gametogenesis. A) Strains expressing either Igo1-myc8 or Igo1S64A-myc8 cells induced to sporulate. Cells were collected at indicated time points and TCA extracts were prepared. Protein samples were loaded on phos-tag or normal SDS-PAGE gels and analysed by western blotting using anti-Myc antibody. B) DNA content in the two cultures was measured by flow cytometry over a period of 8 hours. (PDF) [file pgen.1004456.s003.pdf]

**A**

|         |      |     |
|---------|------|-----|
| Igo1    | RRIS | 105 |
| Igo2    | RRMS | 106 |
| ENSA    | RKPS | 109 |
| ARPP-19 | RKS  | 104 |

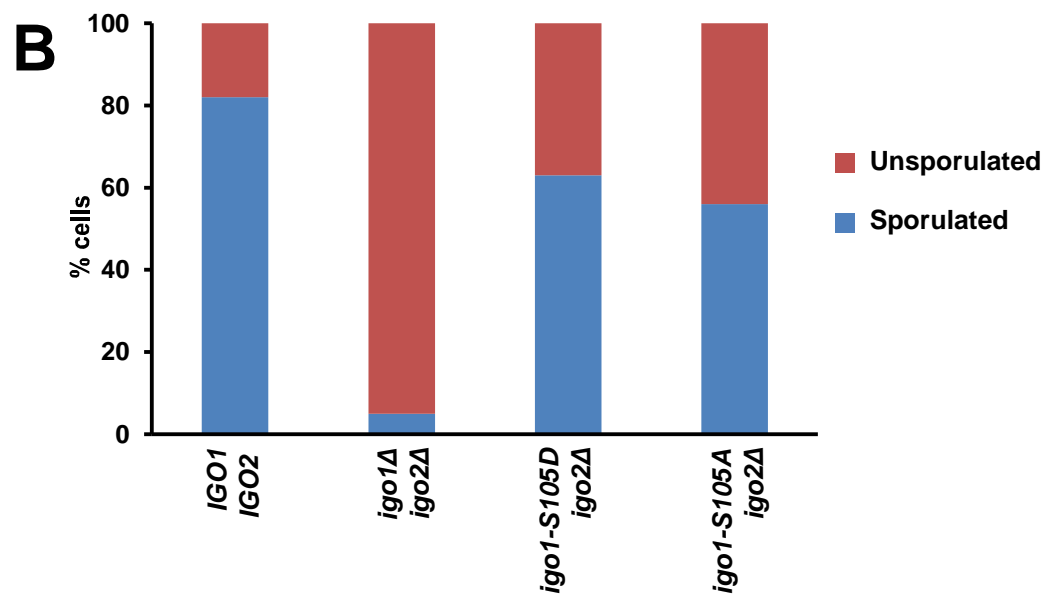

**Figure S4**

Supplement: Figure S4 — The conserved PKA site in Igo1 is dispensable for entry into gametogenesis. A) Conserved PKA site at the C-termini of budding yeast endosulfines Igo1, Igo2 and human endosulfines ENSA and ARPP-19. B) Wild type, igo1Δ igo2Δ, igo1-S105D igo2Δ and igo1-S105A igo2Δ cells were incubated on sporulation plates for 24 hours and the number of sporulated (includes monad, dyad, Tri-/terads) and unsporulated cells were counted using a light microscope. (PDF) [file pgen.1004456.s004.pdf]

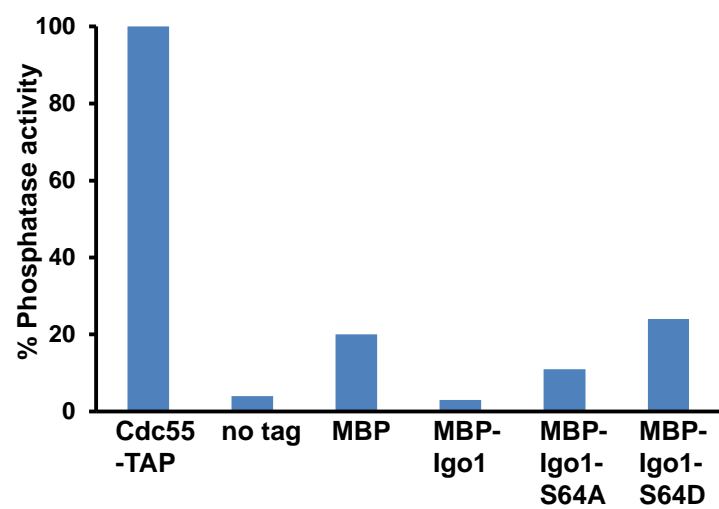

**Figure S5**

Supplement: Figure S5 — Purified endosulfine has no phosphatase activity. 25 µg of purified MBP, MBP-Igo1, MBP-Igo1S64A and MBP-Igo1S64D was incubated with 500 µM phosphopeptide (Millipore). The release of free phosphate was measured using a colorimetric assay (Millipore). TAP eluates from CDC55-TAP and untagged strains were used as positive and negative controls respectively for the phosphatase assay. (PDF) [file pgen.1004456.s005.pdf]

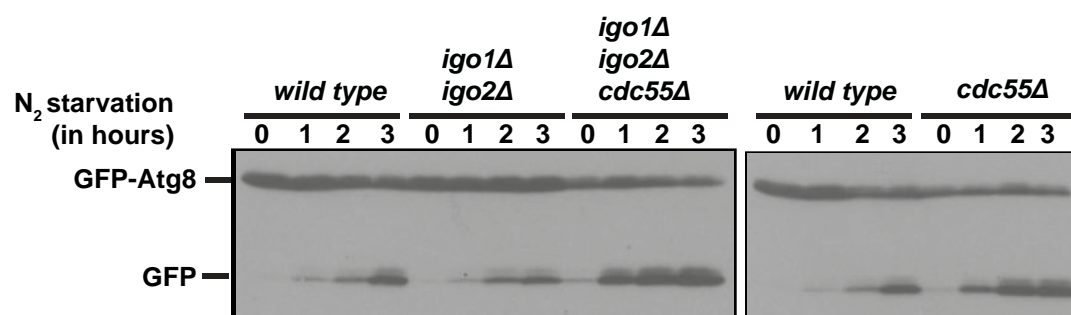

**Figure S6**

Supplement: Figure S6 — Endosulfines are not required for autophagy induced by nitrogen starvation. Wild type, cdc55Δ, igo1Δ igo2Δ and igo1Δ igo2Δ cdc55Δ cells expressing GFP-Atg8 were grown to log phase in YEPD and then transferred to nitrogen deprivation medium. The cultures were incubated further for 3 hours. Cells were collected at indicated time points, total protein extract was prepared and immunoblot analysis was performed using anti-GFP antibody. (PDF) [file pgen.1004456.s006.pdf]

**A**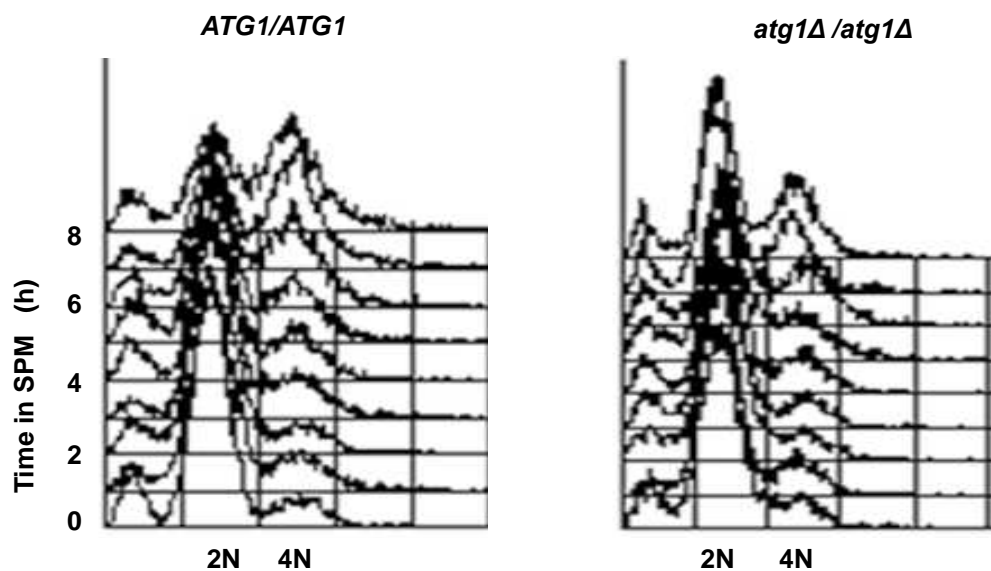**B**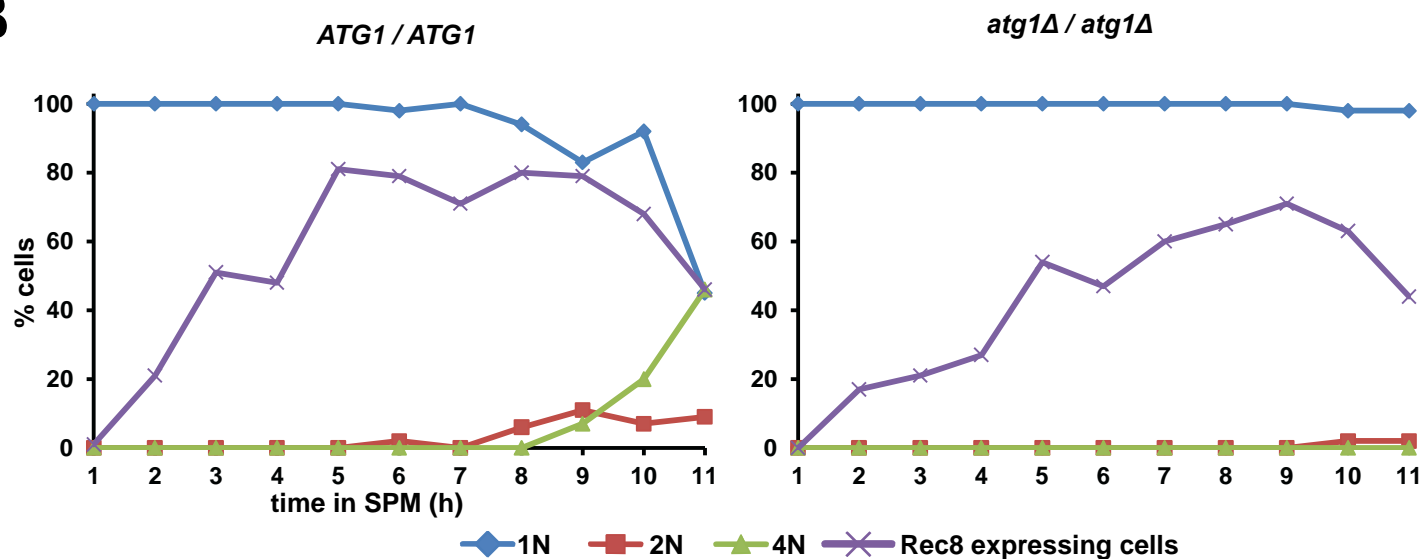**C**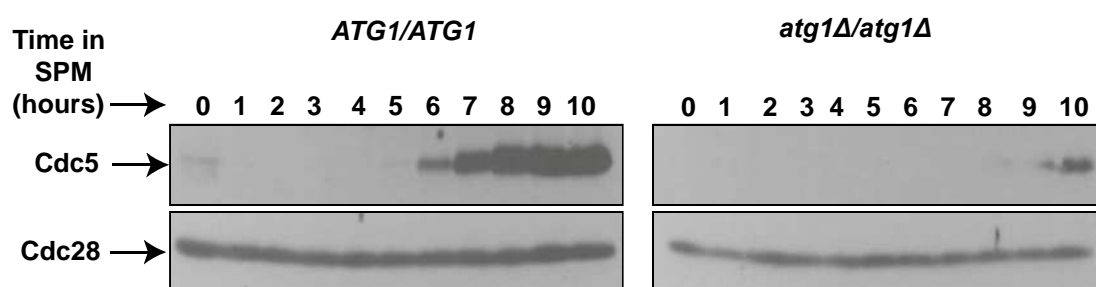**Figure S7**

Supplement: Figure S7 — The atg1Δ strains enter gametogenesis but fail to undergo any meiotic nuclear divisions. Wild-type and atg1Δ cells were induced to enter meiosis by transferring them to SPM. A) Pre-meiotic DNA replication in the cultures was assayed by flow cytometry. B) Kinetics of nuclear division of cells was measured after staining cells with DAPI (n = 100). Rec8 expression was monitored by in situ immunofluorescence using an anti-HA antibody. C) Whole-cell extracts from meiotic cultures taken every hour from 0–10 hours was prepared by TCA method. Protein samples were run on 10% SDS-PAGE, transferred to nitrocellulose membrane and probed with anti-Cdc5 and Cdc28 antibody respectively. (PDF) [file pgen.1004456.s007.pdf]

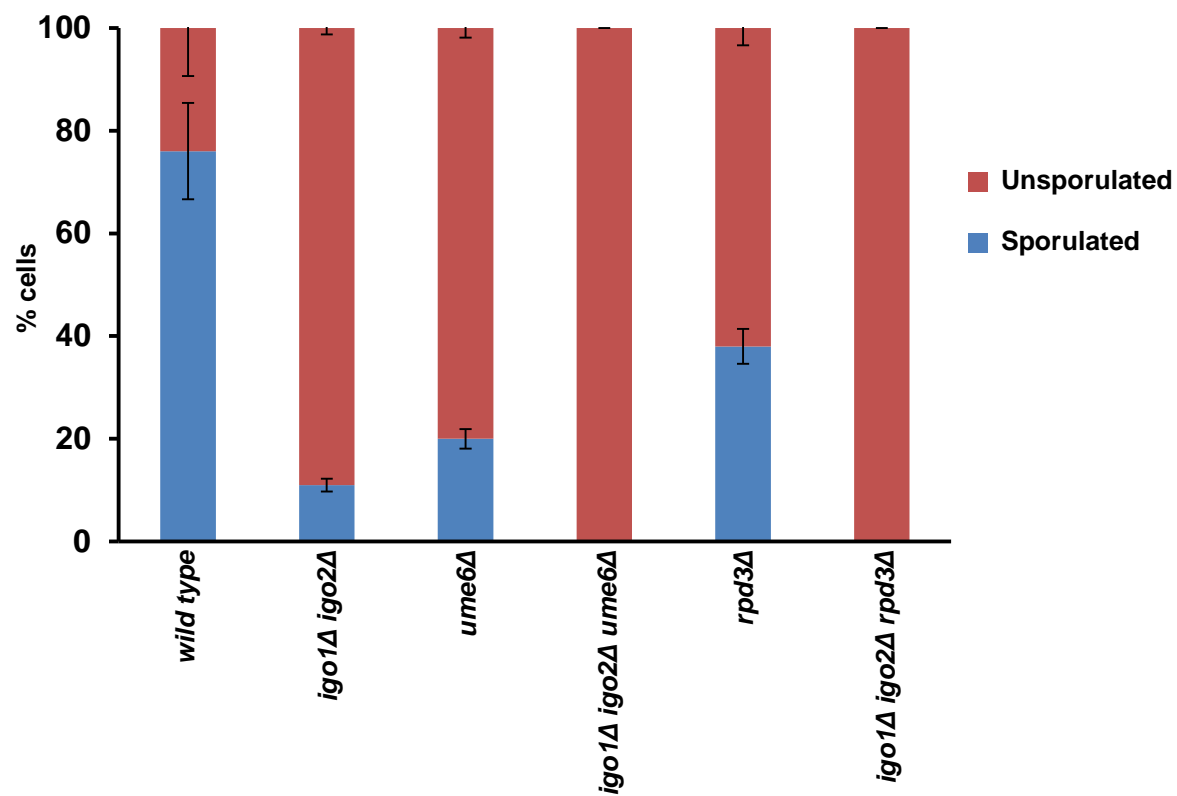

**Figure S8**

Supplement: Figure S8 — ume6Δ and rpd3Δ do not suppress the sporulation defect of igo1Δ igo2Δ cells. Wild-type, ume6Δ, rpd3Δ, igo1Δ igo2Δ, igo1Δ igo2Δ ume6Δ and igo1Δ igo2Δ rpd3Δ cells were incubated for 24 hours on sporulation plates and number of sporulated (includes monad, dyad, Tri-/tetrads) and unsporulated cells were counted using a light microscope. The experiment was repeated 3 times and 200 cells were counted every time for each strain. (PDF) [file pgen.1004456.s008.pdf]
