# Supplementary material for: The Rim15-Endosulfine-PP2ACdc55 Signalling Module Regulates Entry into Gametogenesis and Quiescence via Distinct Mechanisms in Budding Yeast
Source: PLoS Genet. 2014 Jun 26;10(6):e1004456. doi: 10.1371/journal.pgen.1004456 (PMC4072559; doi:10.1371/journal.pgen.1004456)
Supplement: Table S1 — List of yeast strains used. All yeast strains are derivatives of SK1 and have the following markers, unless otherwise stated. ho::LYS2/ho::LYS2, ura3/ura3, leu2::hisG/leu2::hisG, trp1::hisG/trp1::hisG, his3::hisG/his3::hisG, lys2/lys2. (PDF) [file pgen.1004456.s009.pdf]

Strain number	Genotype	Used in Figure
1738	*SK1 MATa/MATα*	1A, 2A, S1, S4
3139	*SK1 MATa/MATα igo1::HphMX6*	1A
3140	*SK1 MATa/MATα igo2::NATMX6*	1A
2473	*SK1 MATa/MATα igo1::HphMX6 igo2::NATMX6*	1A, 1F, 2A, 4A, 7C, S1, S4
2535	*SK1 MATa/MATα* P_{URA3}*::tetR::GFP::LEU2 REC8-ha3::URA3 PDS1-myc18::TRP(K.lactis) IME1-pk6::HIS3 ura3/ura3:: tetOx224-URA3*	1B-D
2538	*SK1 MATa/MATα* P_{URA3}*::tetR::GFP::LEU2 REC8-ha3::URA3 PDS1-myc18::TRP(K.lactis) IME1-pk6::HIS3 igo1::HphMX6 igo2::NATMX6 ura3/ura3:: tetOx224-URA3*	1B-D
2917	*SK1 MATa/MATα igo1:HphMX6-IGO1-myc8-HIS3 igo2::NATMX6*	1F, S2
2918	*SK1 MATa/MATα igo1:HphMX6-igo1S64A-myc8-HIS3 igo2::NATMX6*	1F , S2
2919	*SK1 MATa/ MATα igo1:HphMX6-igo1S64D-myc8-HIS3 igo2::NATMX6*	1F, S2
2921	*SK1 MATa/MATα igo1:HphMX6-igo1S64D-myc8-*	S2

	HIS3 igo2::NATMX6 rim15::HIS3MX6	
1737	*SK1 MATa/MATα CDC55::P$_{CLB2}$-ha3-CDC55:KanMX6*	2A
2745	*SK1 MATa/MATα igo1::HphMX6 igo2::NATMX6 CDC55::P$_{CLB2}$-ha3-CDC55:KanMX6*	2A, 4A
2726	*SK1 MATa/MATα igo1::HphMX6 igo2::NATMX6 CDC55::P$_{CLB2}$-CDC55:KanMX6 net1::HIS3MX6 trp1:net1-6CDK-TEV-myc9::TRP1*	2A
3023	*SK1 MATa/MATα igo1::HphMX6 igo2::NATMX6 CDC55::P$_{CLB2}$-ha3-CDC55:KanMX6 net1::HIS3MX6 trp1:net1-6CDK-TEV-myc9::TRP1*	2A
2989	*SK1 MATa/MATα rts1::NATMX4*	2A
2990	*SK1 MATa/MATα igo1::HphMX6 igo2::NATMX6 rts1::NATMX4*	2A
3028	*SK1 MATa/MATα rim15::HIS3MX6*	2A and S2
3029	*SK1 MATa/MATα rim15::HIS3MX6, CDC55::P$_{CLB2}$-ha3-CDC55:KanMX6*	2A
2796	*SK1 MATa/MATα rim15::HIS3MX6 CDC55::P$_{CLB2}$-CDC55:KanMX6 net1::HIS3MX6 trp1:net1-6CDK-TEV-myc9::TRP1*	2A
2801	*SK1 MATa/MATα rim15::HIS3MX6 CDC55::P$_{CLB2}$-ha3-CDC55:KanMX6 net1::HIS3MX6 trp1:net1-TEV-myc9::TRP1*	2A
3009	*SK1 MATa/MATα ura3::GFP-ATG8-URA3*	2B-C, 6A, 7A-B,

		S6
3011	*SK1 MATa/MATα igo1::HphMX6 igo2::NATMX6 ura3::GFP-ATG8-URA3*	2B-C,6A, 7A-B, S6
3012	*SK1 MATa/MATα igo1::HphMX6 igo2::NATMX6 , CDC55::P*$_{CLB2}$*ha3-CDC55:KANMX6 ura3::GFP-ATG8-URA3*	2B-C, 7A
3213	*SK1 MATα ura3::HSP26-ha3-URA3*	2D, 4B, 5B
3214	*SK1 MATα igo1::HphMX6 igo2::NATMX6 , ura3::HSP26-ha-URA3*	2D, 5B
3215	*SK1 MATα cdc55::NATMX6 ura3::HSP26-ha3-URA3*	2D, 4B
3216	*SK1 MATα igo1::HphMX6 igo2::NATMX6 cdc55::NATMX6 ura3::HSP26-ha3::URA3*	2D, 4B
1771	*SK1 MATa/MATα cdc55::CDC55-TAP::TRP1*	3A
1768	*SK1 MATα cdc55::CDC55-TAP::TRP1*	3B-E, S5
1151	*SK1 MATα*	3C- D, S5
2965	*W303 MATa leu2 ura3-52 prb1-1122 pep4-3 prc1-407 gal2*	3B
2803	*SK1 MATa/MATα igo1::HphMX6 igo2::NATMX6 dhh1::HIS3MX6*	4A

2857	*SK1 MATa/MATα igo1::HphMX6 igo2::NATMX6 ccr4::KANMX6*	4A
3217	*SK1 MATα igo1::HphMX6 igo2::NATMX6 dhh1::HIS3MX6 ura3::HSP26-ha3-URA3*	4B
3218	*SK1 MATα igo1::HphMX6 igo2::NATMX6 ccr4::KANMX6 ura3::HSP26-ha3-URA3*	4B
3211	*SK1 MATa/MATα msn2::HIS3MX6 msn4::NATMX6 gis1::HIS3MX6*	5A
3238	*SK1 MATa/MATα msn2::HIS3MX6 msn4::NATMX6 gis1::HIS3MX6 igo1::HphMX6 igo2::NATMX6*	5A
3239	*SK1 MATa/MATα igo1::HphMX6 igo2::NATMX6*	5A
3257	*SK1 MATa/MATα msn2::HIS3 msn4::NATMX6 gis1::HIS3MX6 ura3::HSP26-ha3::URA3*	5B
2988	*SK1 MATa/MATα leu2::GAL4-ER::LEU2* P_{GAL1}*-IME1::KANMX6*	6B-D
2987	*SK1 MATa/MATα igo1::HphMX6 igo2::NATMX6* P_{GAL1}*-IME1::KANMX6 leu2:GAL4-ER::LEU2*	6B-D
3010	*SK1 MATa/MATα CDC55::*P_{CLB2}*ha3-CDC55:KANMX6 ura3::GFP-ATG8-URA3*	7A
2923	*SK1 MATa/MATα igo1:HphMX6 igo2:NATMX6*	7B,S5

	cdc55::NATMX6 ura3::GFP-ATG8-URA3	
3240	*SK1 MATa/MATα cdc55::NATMX6 ura3::GFP-ATG8-URA3*	7B,S5
2392	*SK1 MATa/MATα*	7C
3179	*SK1 MATa/MATα igo1::HphMX6-IGO1myc8-HIS3*	S3
3180	*SK1 MATa/MATα igo1::HphMX6-igo1S64A-myc8-HIS3*	S3
3141	*SK1 MATa/MATα igo1:HphMX6-igo1S105D-myc8-HIS3 igo2::NATMX6*	S4
3135	*SK1 MATa/MATα igo1:HphMX6-igo1S105A-myc8-HIS3 igo2::NATMX6*	S4
3273	*SK1 MATa/MATα atg1::HphMX6* P_{URA3}*::tetR::GFP-LEU2 tetOx224-URA3REC8-ha3::URA3 PDS1-myc18::TRP1(K.lactis)*	S7
1005	*SK1 MATa/MATα* P_{URA3}*::tetR::GFP-LEU2 tetOx224-URA3 REC8-ha3::URA3 PDS1-myc18::TRP1(K. lactis)*	S7
3260	*SK1 MATa/MATα ume6::HIS3MX6*	S8
3263	*SK1 MATa/MATα ume6::HIS3MX6 igo1::NATMX6 igo2::HphMX6*	S8
3280	*SK1 MATa/MATα rpd3Δ::KANMX4*	S8

3283	*SK1 MATa/MATα igo1::HphMX6 igo2::NATMX6 rpd3Δ::KANMX4*	S8
